# PhoPR variants from several phylogenetic lineages of tuberculosis bacilli respond differently to extracellular signals

**DOI:** 10.1128/mbio.00939-26

**Published:** 2026-05-29

**Authors:** Eva Meunier, Carlos Adriano de Matos e Silva, Wladimir Malaga, David Rengel, Lionel Mourey, Catherine Astarie-Dequeker, Christophe Guilhot

**Affiliations:** 1 Institut de Pharmacologie et de Biologie Structurale (IPBS), Université de Toulouse, CNRS, UPS 27091, Toulouse, France; NYU Langone Health 12297, New York, New York, USA

**Keywords:** PhoPR, two-component regulatory systems, *Mycobacterium tuberculosis*, polymorphism, virulence

## Abstract

The global spread of *Mycobacterium tuberculosis* (*MTB*) is facilitated by airborne transmission, a low infectious dose, and the pathogen’s ability to persist in a latent state. Despite sharing over 99% genomic identity, *MTB* strains and lineages show significant variability in transmissibility. We hypothesized that natural polymorphisms in the two-component regulatory system (TCS) PhoP-PhoR (PhoPR), a major virulence regulator, contribute to this variability.

First, we identified zinc and cadmium as new inducers of PhoPR. Second, we expressed *phoPR* variants from strains with varying transmissibility in the *MTB* HN878 strain and compared the variants’ capacity to induce the expression of PhoP-controlled genes (PhoPR activity) after being submitted to various chemical or cellular signals. *In vitro*, PhoPR systems from HN878 and the low-transmissibility strain *M. canettii* STB-D responded similarly to acidic and zinc stresses. In contrast, PhoPR variants from strains *M. canettii* STB-J and *M. bovis* AN5 showed reduced responsiveness. However, the former seems constitutively activated, whereas the latter displays low activity. Interestingly, although the variant found in *M. bovis* B, which was responsible for direct transmission in humans in contrast to most *M. bovis* strains, did not respond to acidic or zinc stress *in vitro*, it remained inducible in macrophages. Finally, we compared the virulence of the various recombinant strains in C3HeB/FeJ mice. We found that only the strain expressing the PhoPR variant from *M. bovis* AN5 is associated with reduced virulence.

These findings indicate that both *phoPR* polymorphisms and the strain-specific genetic context modulate PhoPR activity, thereby influencing *MTB* virulence and potentially its transmissibility.

**IMPORTANCE:**

Understanding genomic adaptations favoring persistence and transmission of tuberculosis bacilli is crucial for developing new strategies to combat tuberculosis. This study explores the impact of natural mutations on the response to environmental cues of the two-component regulatory system (TCS) PhoP-PhoR (PhoPR), which controls the expression of major virulence factors, and on the virulence of *Mycobacterium tuberculosis* (*MTB*). Our findings identify new signals activating PhoPR and provide new insights into the molecular mechanisms of PhoPR. In addition, this study highlights how *phoPR* polymorphisms may influence the epidemic capacity of tuberculosis bacilli.

## INTRODUCTION

*Mycobacterium tuberculosis* (*MTB*), the main causative agent of human tuberculosis (TB), emerged as a professional pathogen from an environmental bacillus between 6,000 and 70,000 years ago (1) and adapted to perform an infectious cycle in humans, without any environmental or animal reservoir. Thanks to its molecular evolution, *M. tuberculosis* can infect and multiply within alveolar macrophages, resist attack by the host immune response, cause tissue lesions and coughing that facilitate the production and release of infectious droplets, and ultimately survive aerosol transportation to a naive host (2). For the most part, adaptations associated with these remarkable capacities remain yet unknown.

TB is a chronic infectious disease caused mostly by *M. tuberculosis* and closely related bacilli, forming the *M. tuberculosis* complex (MTBC) and sharing the same ancestor (genome sequences among the MTBC group display an average nucleotide identity greater than 99%). The MTBC comprises human-adapted lineages, such as *Mycobacterium tuberculosis sensu stricto* (*MTB*) and *Mycobacterium africanum*, as well as animal-adapted lineages, such as *Mycobacterium bovis* (3–5). In addition, few TB cases are due to infection by bacteria of the *Mycobacterium canettii* group, which is more distantly related to the MTBC. The *M. canettii* strains have never been reported to be involved in transmission chains among humans, with extremely limited contribution to the TB pandemic, and they might represent opportunistic pathogens, with environmental reservoirs, phenotypically close to the ancestor of MTBC (6, 7).

Comparative genomic analyses revealed that the actual MTBC bacilli have evolved clonally from a unique ancestor without the acquisition of exogenous DNA by horizontal gene transfer (1). Due to the absence of horizontal gene transfer, chromosomal mutations are the main source of genetic diversity among strains of the MTBC. Interestingly, recent studies have shown that some genes in MTBC clinical isolates display a higher frequency of nonsynonymous mutations (dN) than synonymous mutations (dS) (dN/dS > 1), indicating that these genes are possibly under positive (adaptive) selection in MTBC (8–10). Among these genes, *phoR* is one of the genes with the highest dN/dS value, which suggests that the *phoR* gene plays an important role in the modern evolutionary history of MTBC.

The *phoR* gene encodes for the sensor protein of the two-component regulatory system (TCS) PhoP-PhoR (PhoPR) required for full virulence of *MTB* (11–14). PhoR is a homodimer that detects and is activated by environmental cues, such as acidic stress (15, 16), through its extracytoplasmic sensor domain. Based on findings in other bacteria, it was proposed that, after activation of PhoR, the signal is propagated across the plasma membrane through conformational changes that switch on the kinase activity of the protein, leading to autophosphorylation of His259 (17). Upon recruitment of PhoP, the response regulator, the phosphoryl group is transferred to PhoP at position Asp71, which enhances its binding affinity to the DNA target sequence and modulates the expression of various genes (18, 19). PhoPR directly or indirectly regulates more than 80 genes, including *espACD* required for the secretion of the major virulence factor EsxA (also known as 6 kDa early secretory antigenic target [ESAT-6]) and biosynthetic genes of surface lipids, such as sulfolipids (SL) or poly-acyltrehaloses (PAT) (13, 14, 20–22), and is considered to be a master regulator of virulence for TB bacilli (14, 23). Moreover, it was recently demonstrated that SL activates nociceptive neurons and induces cough in the guinea pig model, suggesting that, through the production of SL, PhoPR may be indirectly a key determinant of TB transmission (24). This hypothesis was supported by a genomic study that showed a positive correlation with single-nucleotide polymorphisms (SNPs) in PhoR and enhanced transmission (25). Previous studies found that SNPs in *phoR* from various MTBC lineages or strains decreased the expression of the PhoP regulon and caused lower virulence in human macrophages and animal models (26–28). These studies strongly support the conclusion that natural mutations found in *phoR* impact the activity of the PhoPR TCS (i.e., the capacity to induce the expression of genes controlled by the TCS) with consequences on host interaction. However, there is a lack of information regarding the impact of *phoR* mutations on the response to signals and activation of the PhoPR TCS.

To address this question, we expressed several PhoPR variants from different MTBC and *M. canettii* phylogenetic lineages in the same *MTB* genetic background. We next assessed how these PhoPR variants respond to various signals in both *in vitro* and *ex vivo* conditions. Finally, we investigated the capacity of the PhoPR variants to affect *MTB* virulence and the formation of lung lesions in C3HeB/FeJ mice, a mouse model that reproduces some traits encountered in humans during TB.

## RESULTS

### Construction of a set of recombinant strains derived from *Mycobacterium tuberculosis* HN878

**Fig 1:**
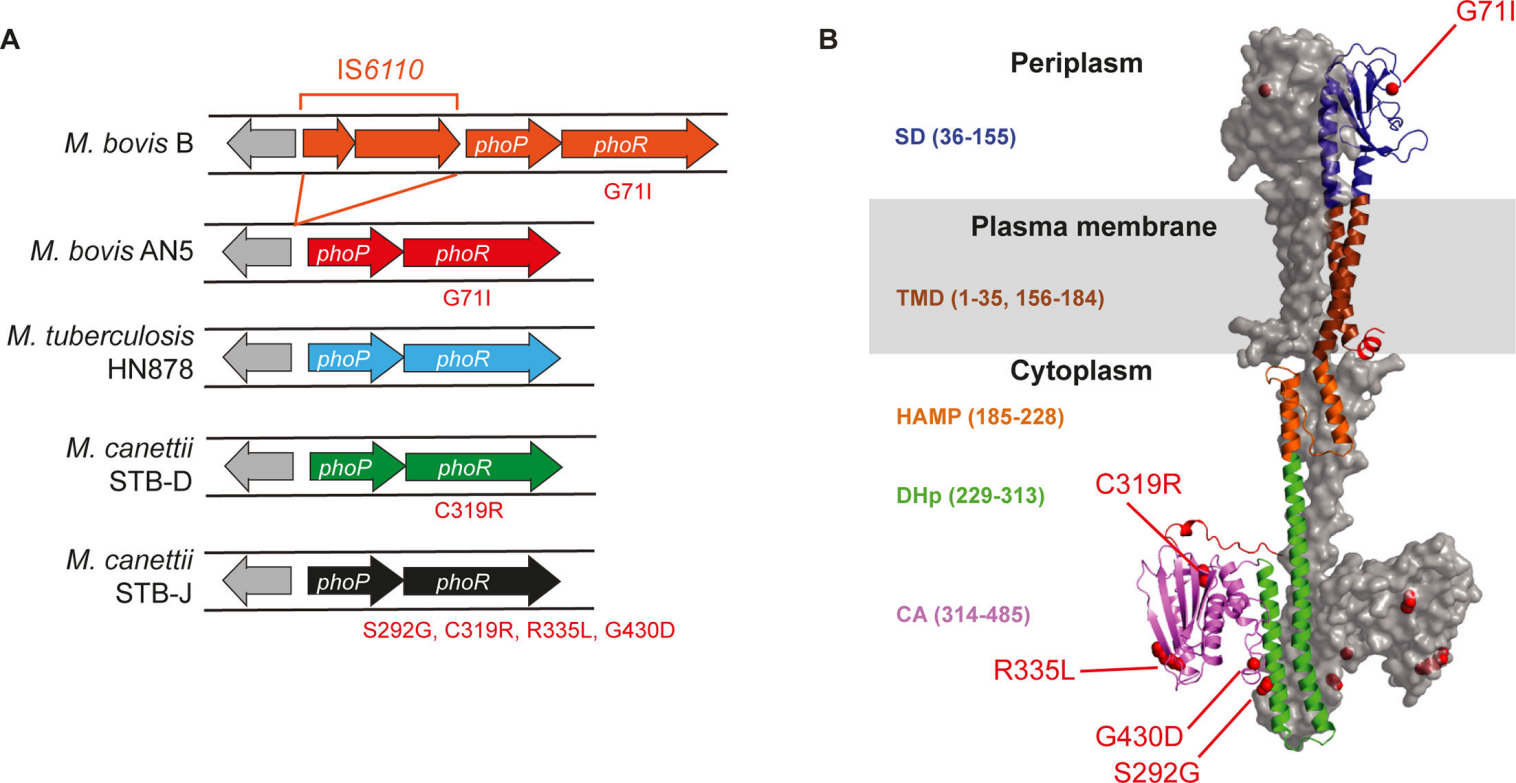
Selected *phoPR* variants and relevant mutations. (**A**) Schematic representation of the *phoPR* locus from five strains belonging to the *Mycobacterium tuberculosis* complex (MTBC) and *M. canettii*, highlighting mutations relative to the *M. tuberculosis* HN878 reference strain. These variants harbor strain- or lineage-specific mutations. We amplified all these variants using specific primers to generate a large 3.3-kb fragment, covering the *phoPR* promoter and *phoPR* genes, that was inserted into the integrative complementation plasmid (see Materials and Methods), and we transferred these constructs into the HN878 Δ*phoPR::km* mutant strain. We also included in this series a recombinant HN878 Δ*phoPR::km* as a control carrying the integrative complementation plasmid without *phoPR*. (**B**) Predicted three-dimensional structure of the PhoR sensor kinase, generated using AlphaFold and visualized with PyMOL (27). One monomer is displayed as a colored cartoon, illustrating the different structural domains, while the second monomer is shown as a gray surface. Mutations specific to each PhoR allele are depicted as red spheres. Regions with low prediction confidence (pLDDT < 70) are highlighted in red. Domain annotations: SD, sensor domain; TMD, transmembrane domain; HAMP, domain found in histidine kinases, adenylyl cyclases, methyl-accepting chemotaxis proteins, and phosphatases; DHp, dimerization and histidine phosphotransfer domain; CA, catalytic ATP-binding domain.

To explore the impact of *phoPR* mutations without confounding factors associated with different genetic background, we generated a new series of recombinant strains derived from the clinical isolate *MTB* HN878 belonging to lineage L2 (29). We first replaced the *phoPR* genes by a kanamycin resistance gene (*km*) to create the strain HN878 Δ*phoPR::km,* also named PMM335 (Fig. S1). Next, we selected five *phoPR* variants from strains associated with high or low transmissibility in humans (Fig. 1A and B) and transferred them independently in the HN878 Δ*phoPR::km* mutant on an integrative plasmid derived from pMV361 and containing a streptomycin resistance gene.

The following strains were selected as the source of *phoPR* variants for the construction of the recombinant strains: (i) *MTB* HN878 (*phoPR-HN878*); (ii) *M. bovis* AN5 strain (*phoPR-AN5*); (iii) *M. bovis B* strain (*phoPR-bovis-B*); (iv) *M. canettii* STB-D strain (*phoPR-STB-D*); (v) *M. canettii* STB-J strain (*phoPR-STB-J*) (Fig. 1A and supplemental material).

**Fig 2:**
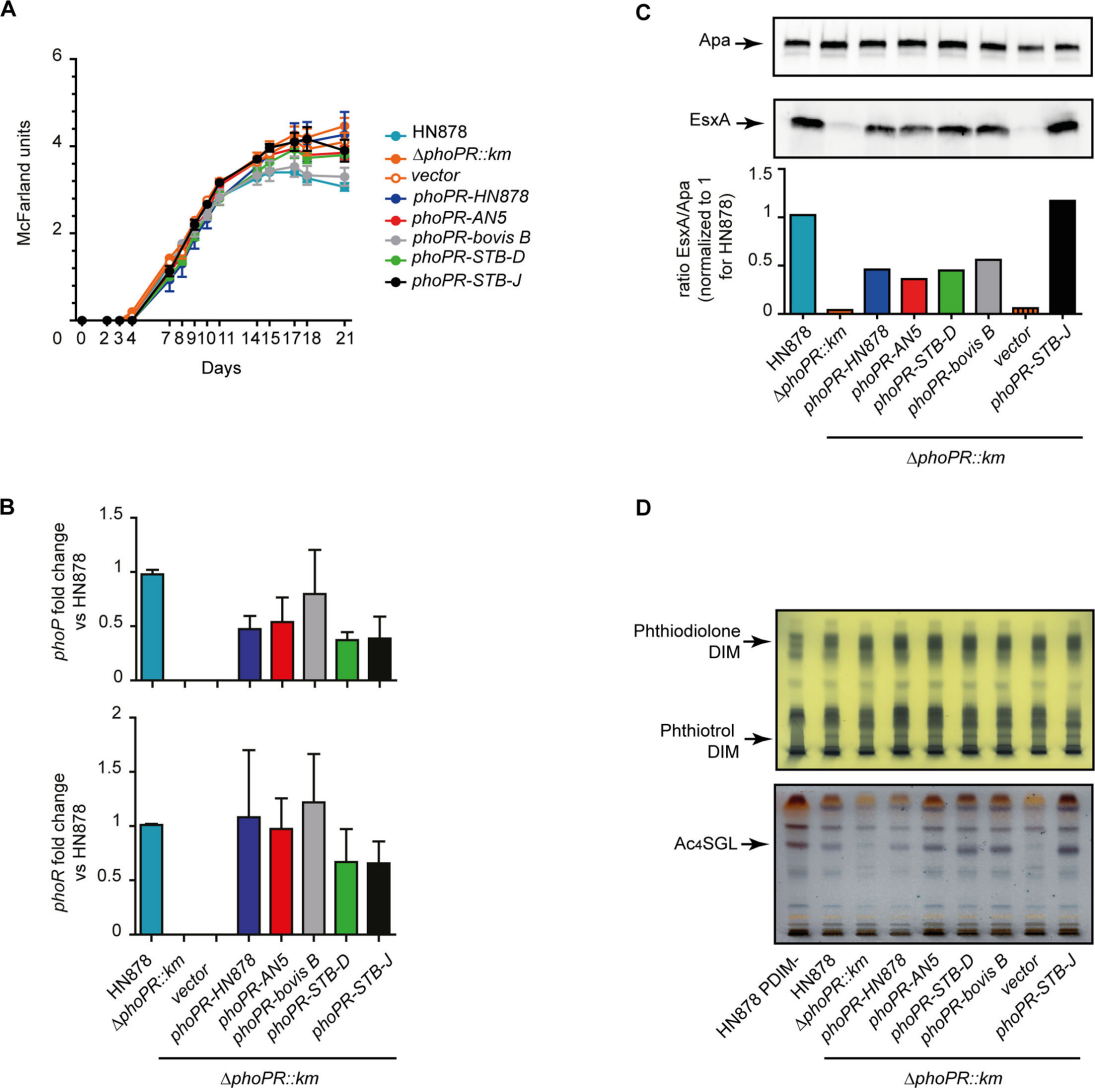
Characterization of the HN878-derived recombinant strains. (**A**) Growth of the various recombinant strains evaluated using turbidimetry (McFarland units) in the standard liquid medium 7H9 ADC Tween 80 0.05%. Values are expressed as mean ± standard deviation from three biological replicates (*n* = 3). (**B**) Relative expression of *phoP* and *phoR* in the recombinant strains in comparison to the parental HN878 WT strain. Gene expression was quantified by RT-qPCR and normalized to *sigA* before calculating the fold change vs HN878 WT. Error bars represent the mean ± standard deviation from three independent experiments (*n* = 3). Recombinant strains express *phoP* and *phoR* at the same level in standard liquid growth conditions. (**C**) Western blot analysis of culture supernatants from recombinant strains, assessing the secretion of EsxA (ESAT-6) and the control protein Apa. Protein extracts were probed with specific anti-EsxA and anti-APA antibodies. Signals were revealed using Imobilon Western chemiluminescent substrate (Millipore) and quantified with the ChemiDoc MP Imaging System (BioRad). As a loading control, we checked the secretion of the antigen APA whose production and secretion are not controlled by PhoP and found no difference between the strains. This experiment was performed once. (**D**) High-performance thin-layer chromatography (HP-TLC) analysis of lipid extracts from recombinant strains. Lipids were dissolved in chloroform at a concentration of 20 mg/mL, and 20 µL of each extract was separated by HP-TLC (Camag) using CHCl₃/CH₃OH/H₂O (60:16:2, vol/vol/vol) for sulfolipids or petroleum ether/diethyl ether (90:10, vol/vol) for PDIM analyses. Lipids were visualized by spraying the plates with anthrone reagent (for sulfolipids) or with 10% phosphomolybdic acid in ethanol (for PDIM), followed by heating. The positions of Ac₄SGL and PDIM are indicated. We used the lipid PDIM as a loading control because its production is not controlled by PhoP. We also used a recombinant HN878 PDIM- strain to precisely localized PDIM. Production of PDIM was similar among the recombinant strains and with the WT HN878 strain. This experiment was performed once.

We first compared their growth in standard liquid medium and their expression of *phoPR* genes and found no significant difference between complemented strains (Fig. 2A and B). However, our experiment cannot exclude that mutations in the *phoPR* variants may impact the protein level. We next analyzed the EsxA secretion and lipid production in this series of HN878 recombinant strains (Fig. 2C and D). As previously described (13, 14, 20–22), the *phoPR* deletion strongly impaired EsxA secretion and production of Ac_4_SGL. Complementation with all the *phoPR* variants restored the antigen secretion and lipid production to a similar level than in the parental HN878 strain. Our data confirmed that we successfully created five recombinant strains expressing *phoPR* variants from various MTBC or *M. canettii* strains.

### Zinc and cadmium in addition to acidic pH and intracellular environment induce a PhoPR-dependent expression of the *aprA*′*::GFP*

To further explore the response of these PhoPR variants to environmental signals, we transferred a fluorescent reporter system into the various HN878 recombinant strains (Fig. S1). This system is an integrative plasmid carrying the *GFP* gene fused to the PhoP-controlled promotor of gene *aprA/mcr7 (aprA*′*::GFP*) and an *mCherry* gene under the *smyc* constitutive promotor (*smyc*′*::mCherry*) derived from pMT-3 (16). The *aprA* and *mcr7* names correspond to the same genetic locus encoding a noncoding regulatory RNA (ncRNA) whose expression is tightly controlled by PhoP (21). We decided to retain the initial name *aprA*′*::GFP* for the fluorescent reporter system (16) and *mcr7* for the ncRNA (21).

**Fig 3:**
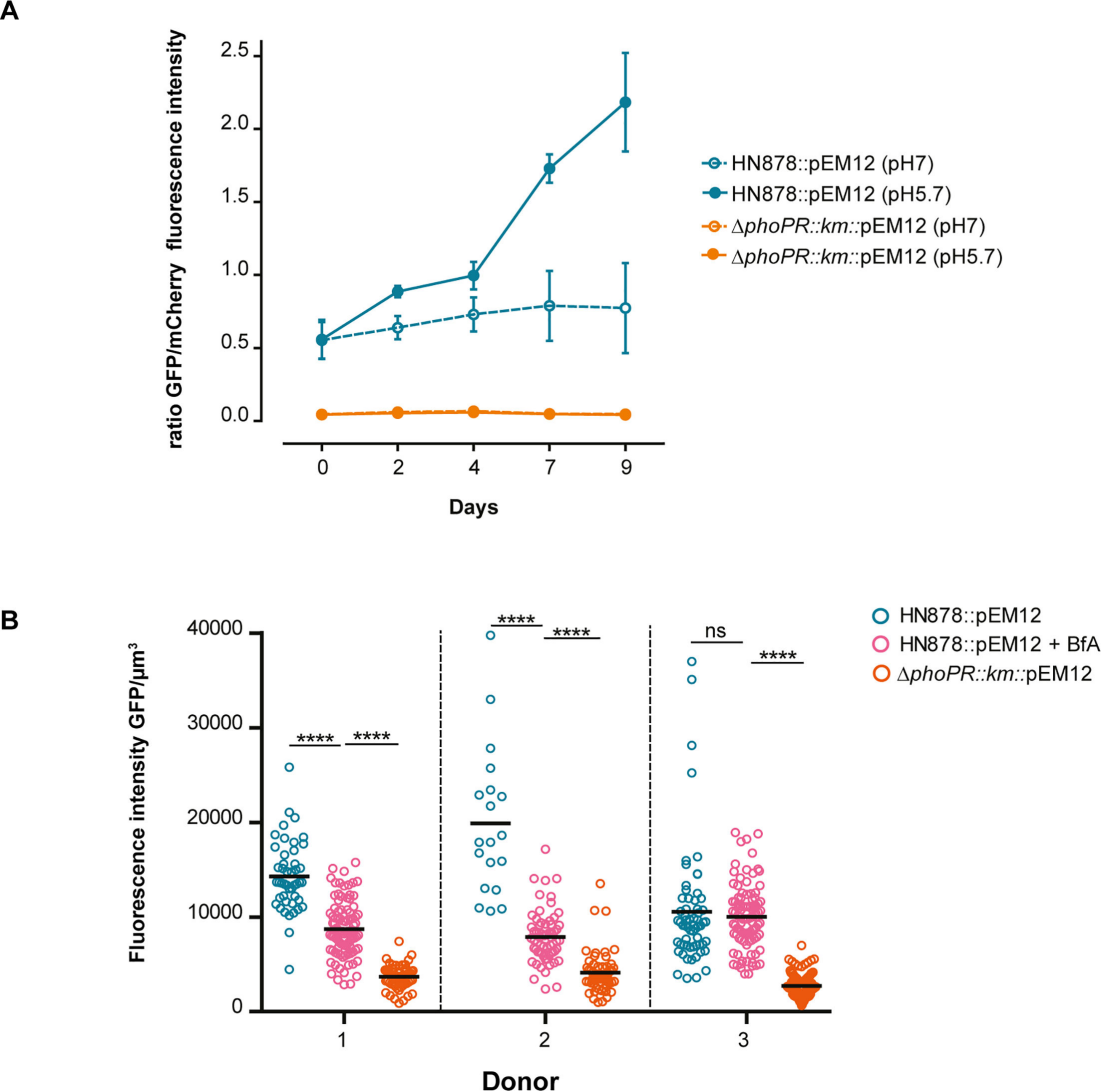
Acidic pH induces activation of the PhoPR system in the HN878 reporter strain (*aprA*′*::GFP,smyc*′*::mCherry*) both *in vitro* and in infected cells. (**A**) Ratio of inducible GFP fluorescence to constitutive mCherry fluorescence (GFP/mCherry) in strains. Solid lines represent fluorescence intensities of strains grown at pH 5.7 (filled circles), while dotted lines represent strains grown at pH 7.0 (open circles). Fluorescence was measured by flow cytometry, with 50,000 bacterial events recorded per sample. Error bars represent the mean ± standard deviation of three independent experiments (*n* = 3). (**B**) Quantification of GFP fluorescence in intracellular bacteria following 24 h of infection, with or without bafilomycin A1 (BfA) treatment. Human monocyte-derived alveolar macrophages (hMDAMs) were infected at a multiplicity of infection (MOI) of 2:1. Infected macrophages were visualized by fluorescence microscopy 24 h post-infection (p.i.) after labeling with CellTracker (BMQC) and fixation. Activation of the PhoPR regulon was assessed by quantifying both intracellular GFP and mCherry signals per bacterium. For each strain, images were acquired from five distinct microscopic fields. Statistical significance was determined using one-way analysis of variance (ANOVA) followed by Bonferroni *post hoc* testing. ****, *P* < 0.0001

Acidic pH was previously found to activate the PhoPR TCS and to induce *aprA*′*::GFP* expression in *MTB* (15, 16). To validate our integrated reporter system, we grew the HN878 strain transformed with the dual fluorescent reporter (*aprA*′*::GFP, smyc*′*::mCherry*) for up to 9 days in 7H9 media with either neutral (pH 7.0) or acidic pH (pH 5.7) and performed a ratiometric analysis of both GFP and mCherry fluorescence over time by flow cytometry (Fig. 3A and Fig. S2). As expected, the mCherry signal remained stable at both pH values in the HN878 (*aprA*′*::GFP, smyc*′*::mCherry*) strain (Fig. S2). The GFP signal also remained stable at neutral pH 7.0, but it increased over time at pH 5.7 (Fig. S2). This increase was PhoPR-dependent as GFP fluorescence remained stable in the HN878 Δ*phoPR::km* (*aprA*′*::GFP, smyc*′*::mCherry*) at both acidic and neutral pH (Fig. 3A and Fig. S2).

We also assessed whether we could detect using our chromosomal reporter system the activation of PhoPR inside human alveolar macrophages. Human monocyte-derived alveolar macrophages (hMDAMs) were infected with either HN878 (*aprA*′*::GFP, smyc*′*::mCherry*) or HN878 Δ*phoPR::km* (*aprA*′*::GFP, smyc*′*::mCherry*) at a multiplicity of infection (MOI) of 2:1. One day post-infection (p.i.), we measured the GFP signal intensity of individual intracellular bacteria using confocal microscopy, as described by Tan and collaborators (15) (Fig. 3B). As previously observed, the distribution of GFP signals in individual intracellular bacteria was highly heterogeneous, consistent with *MTB* encountering various intracellular environments and *aprA-/mcr7*-inducing conditions (16). Both the GFP signal intensity and dispersion were substantially higher for the HN878 strain than for the HN878 Δ*phoPR::km* mutant, indicating that a high GFP fluorescent signal was mostly dependent on PhoPR activation. As PhoPR is activated by acidic pH, we next sought to block phagosomal acidification to test whether this impacts the GFP signal. We treated the hMDAMs with bafilomycin A, a vacuolar proton ATPase inhibitor that acidifies the *MTB*-containing phagosome (30). We then infected the cells with HN878 (*aprA*'*::GFP, smyc*'*::mCherry*) and measured the GFP fluorescence intensity in individual bacteria 1 day p.i. As expected, when cells were treated with bafilomycin A, we found a drop in the intensity of the GFP signal in the intracellular bacteria in two of the three donors compared with untreated conditions (Fig. 3B). This suggests that blocking of phagosomal acidification leads to lower activation of the PhoPR system. However, the signal remains higher than for the HN878 Δ*phoPR::km* strain in untreated hMDAMs, suggesting that the PhoPR system is not completely switched off. Taken together, these results confirm previous observations made in a different *MTB* background (16) and demonstrate that the chromosomally integrated reporter system is suitable for detecting PhoPR activation.

**Fig 4:**
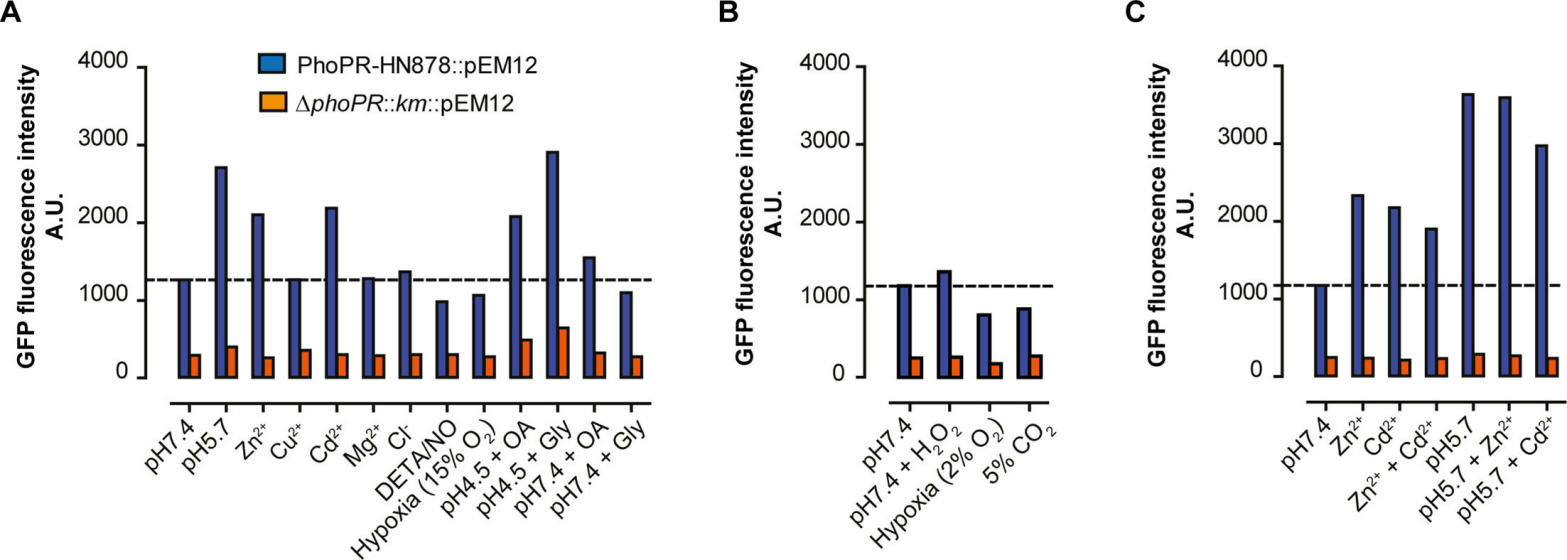
In addition to acidic pH, the *phoPR* variant from the HN878 strain (PhoPR-HN878::pEM12) is also activated upon zinc and cadmium exposure. (**A through C**) Inducible GFP fluorescence intensity of recombinant strains following a 4-day exposure to various stress conditions. The black dotted line represents the baseline activation of the PhoPR system in the reporter strain PhoPR-HN878::pEM12 under non-stress conditions (pH 7.4). Unless otherwise indicated in the figure, all tested conditions were adjusted to pH 7.4. When not specified in the graph, stress agents were used at the following concentrations: Zn^2+^ (ZnSO₄, 500 µM), Cu^2+^ (CuSO₄, 500 µM), Cd^2+^ (CdSO₄, 200 µM), Cl^−^ (NaCl, 500 mM), DETA/NO (500 µM), Gly (glycerol, 0.2%), and OA (oleic acid, 200 µM). Fluorescence was quantified by flow cytometry, with 50,000 bacterial events recorded per sample. We did not detect any induction of *aprA*′:*:GFP* upon incubation of the recombinant HN878 strain with 500 mM NaCl, consistent with the lack of *aprA/mcr7* induction found upon incubation of *MTB* with 250 mM NaCl (15). This result contrasts with the high rate of PhoR phosphorylation found in high-NaCl conditions (35). One possible explanation is the use of PhoR from H37Rv in the latter study. This variant possesses a P172L mutation that is specific to H37Rv and increases the basal PhoR activity (36). Each panel corresponds to a single experiment.

These results also prompted us to test other potential signals, such as various cations present in the phagosomal vacuole, oxidative and nitrosative stress, hypoxia, and oleic acid, that *MTB* encounters during its intracellular growth in addition to acidic pH (31–34) (Fig. 4A through C). In addition to acidic pH, we found that Zn^2+^ and Cd^2+^ were able to increase the GFP signal, and this effect was PhoPR-dependent. Finally, we found that the combination of inducing cues (Zn^2+^ + Cd^2+^, Zn^2+^ + acidic pH, and Cd^2+^ + acidic pH) did not further enhance the GFP fluorescence over the independent signals, indicating no synergistic effect on PhoPR activity (Fig. 4C and Fig. S3). These results establish that Zn^2+^ and Cd^2+^, in addition to acidic pH, are activators of the PhoPR system.

### PhoPR variants display different responses to acidic pH, zinc, and in macrophages

**Fig 5:**
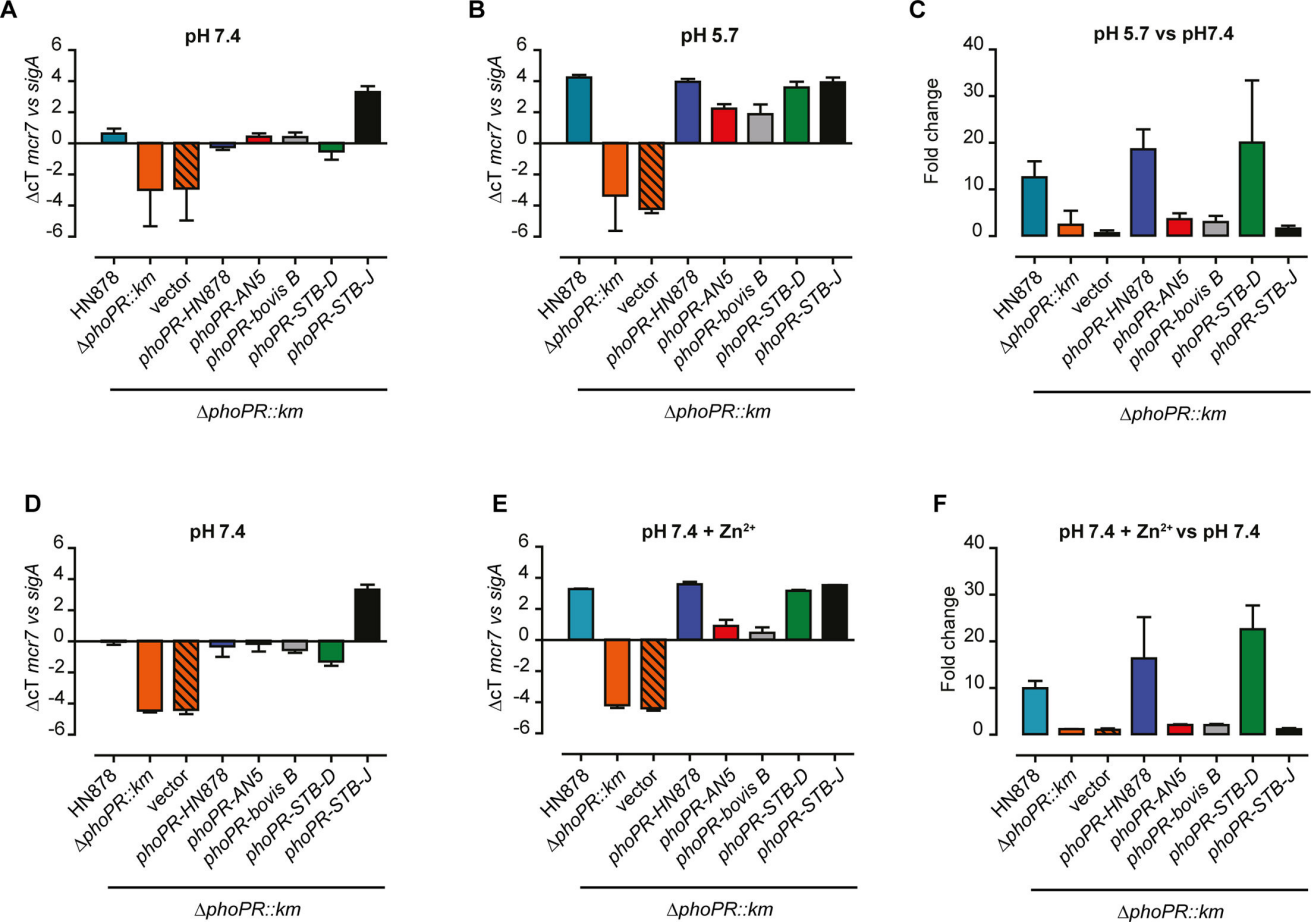
The *phoPR* variants respond differently to acidic pH and zinc. (**A through F**) Relative expression of the *mcr7* gene, normalized to the housekeeping gene *sigA*, in WT HN878, Δ*phopR::km* mutant or recombinant strains expressing PhoPR variants. Gene expression was measured after exposure to acidic pH (pH 5.7) (**B**), zinc stress (ZnSO₄, 500 µM) (**E**), or non-stress conditions (pH 7.4) (**A and D**). The induction of *mcr7* under stress conditions is shown as fold change relative to the baseline expression observed at pH 7.4 (**C and F**). Gene expression was quantified by RT-qPCR. Error bars represent the mean ± standard deviation from three independent experiments (*n* = 3).

Next, we sought to compare the response of the PhoPR variants to inducing signals or environments. First, we exposed the HN878 series of recombinant strains to 7H9 medium at pH 7.4 with low zinc concentration, 7H9 with acidic pH (pH 5.7) and with low zinc concentration, and 7H9 (pH 7.4) with low or high zinc concentrations (500 µM). We monitored the expression of *aprA/mcr7* indirectly using the GFP fluorescence intensity (Fig. S4) and directly by RT-qPCR (Fig. 5) as indicators of PhoPR activity.

Our data showed that the lack of *phoPR* strongly impaired the basal level of *aprA/mcr7* expression in standard medium (pH 7.4 and low zinc concentration) and incubation at acidic pH or high zinc concentration did not induce *aprA*/*mcr7* (Fig. S4 and Fig. 5). The complementation with *phoPR-HN878* partially restored the *aprA/mcr7* level in comparison to the WT level in standard 7H9 medium. The GFP fluorescence or *mcr7* RNA level increased strongly at acidic pH or high zinc concentration, and the induction vs standard 7H9 was similar to that in HN878 WT (Fig. 5 and Fig. S4). In contrast, whereas complementation with *phoPR* variants from *M. bovis* AN5 or *M. bovis* B gave the basal expression level of *aprA*/*mcr7* similar to that of *phoPR-HN878,* both acidic pH and high zinc concentration only slightly enhanced this level (Fig. 5 and Fig. S4). Finally, complementation with the *phoPR* variants from *M. canettii* gave opposite results. On the one hand, the *phoPR-STB-D* restored basal *aprA/mcr7* expression and induction similar to that of *phoPR-HN878*. On the other hand, the strain complemented with *phoPR-STB-J* displayed a higher basal expression than the *phoPR-HN878-*complemented strain in standard 7H9 conditions, but acidic pH and high zinc failed to enhance the *mcr7* RNA level or GFP fluorescence. These results were not restricted to *aprA*/*mcr7* since we observed similar results for the expression of *pks2*, another gene under the control of PhoPR in *MTB*, during growth at neutral or acidic pH (Fig. S5). We finally addressed whether these differences are conserved in native strains by analyzing *mcr7* expression after growth at pH 7.4 or 5.7 (Fig. S6). We tested four out of five native strains (*M. bovis* B was not evaluated) and found similar *mcr7* induction than in the recombinant strain expressing the corresponding PhoPR variants for three native strains. The only exception was *M. canettii* STB-D, for which we observed no *mcr7* induction at acidic pH, suggesting an influence of the genetic background (Fig. S6).

**Fig 6:**
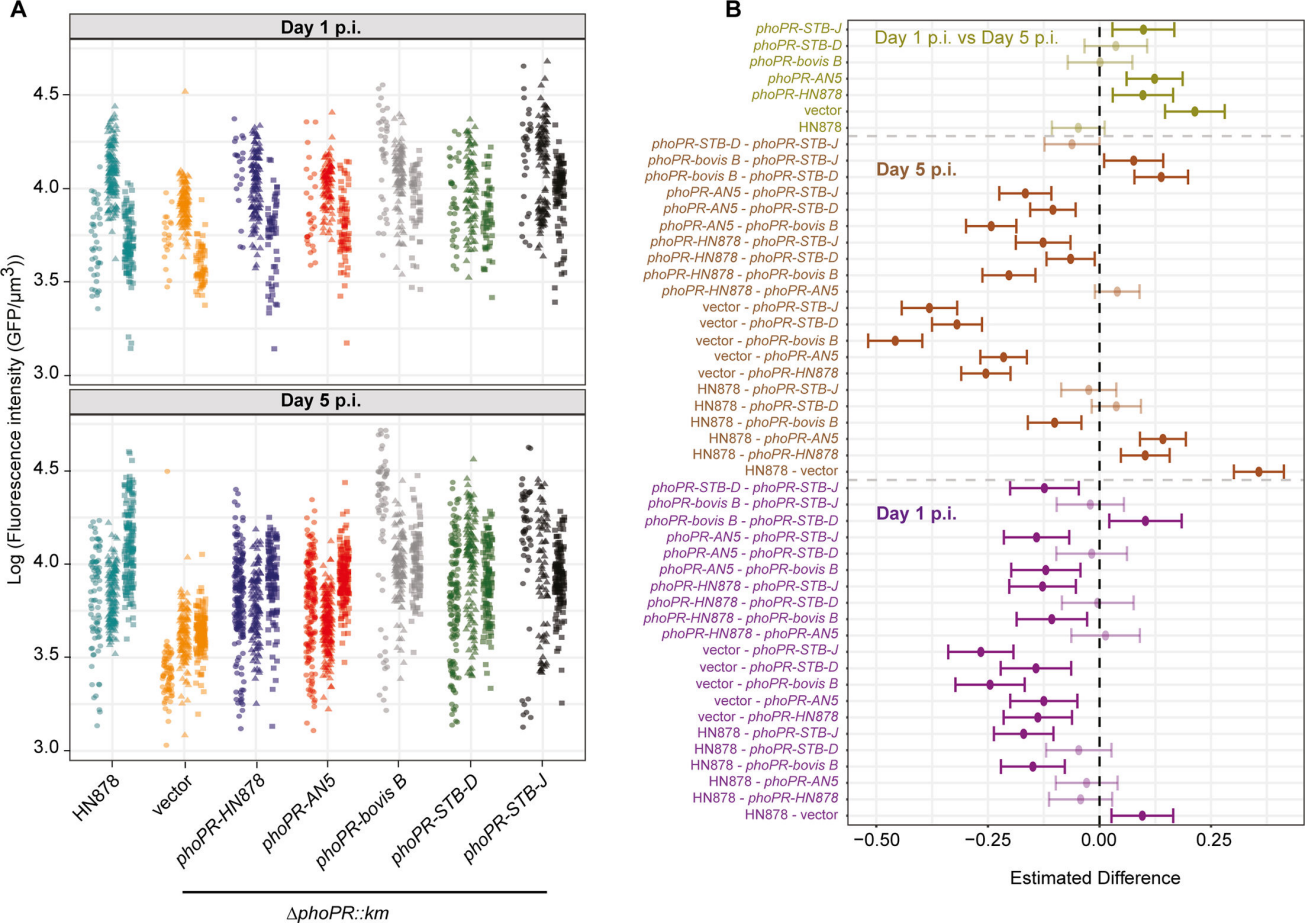
*aprA*′*::GFP* expression in bacteria expressing various *phoPR* variants during human macrophage infection. hMDAMs derived from three independent donors (circle, triangle, and square symbols) were infected with the wild-type HN878, the mutant strains complemented with either the empty vector, or the mutant strains complemented with various *phoPR* variants. (**A**) GFP fluorescence intensity of individual intracellular recombinant bacteria was determined by fluorescent microscopy at 1 or 5 days p.i. Each dot corresponds to a single bacterium. (**B**) Statistical analyses of the data from panel **A**, comparing results from days 1 and 5 for each strain (green upper part) and between a pair of strains at days 1 and 5 p.i. (violet lower and brown medium parts, respectively). Statistical analyses were performed as described in Material and Methods. Estimated difference and 95% confidence intervals (CI) are shown. Comparisons for which the absence of difference falls outside the CI are, therefore, considered as significant (full-color traits vs shaded traits, i.e., nonsignificant). Greater distance of the CI from the absence of difference implies higher significance. Differences reaching statistical significance are indicated using a dark line, whereas nonsignificant differences are indicated using shaded lines.

Next, we compared the activity of PhoPR variants in the intracellular environment of hMDAMs. To this end, cells were infected with either the HN878 WT, the Δ*phoPR::km* mutant, or the strains expressing the *phoPR* variants and carrying the *aprA*′*::GFP, smyc*′*::mCherry* reporter system, and we monitored GFP expression in individual intracellular bacteria at days 1 and 5 p.i. (Fig. 6). As expected, we observed a higher fluorescence intensity in all recombinant strains expressing *phoPR* variants in comparison to the Δ*phoPR::km* strain (Fig. 6A and B). The comparative analysis of the GFP intensity in the complemented strains revealed that, at 1 day p.i, the distributions were similar for the strains expressing *phoPR-HN878*, *phoPR-AN5,* and *phoPR-STB-D* but shifted to higher values for those expressing *phoPR-bovis B* and *phoPR-STB-J*, which were similar. At 5 days p.i, we found that the strain expressing *phoPR-bovis B* displayed significantly higher fluorescence than all the other complemented strains; the strain *phoPR-STB-J* gave similar signal intensities than the strain *phoPR-STB-D,* but these intensities were higher than those for *phoPR-HN878* and *phoPR-AN5; phoPR-HN878* and *phoPR-AN5* gave similar fluorescence signals. These differences were also observed when analyzing the fluorescence intensity intervals (Fig. S7).

Overall, we identified four different behaviors among the PhoPR variants regarding their response to environmental cues: (i) PhoPR-AN5 seems to be the least active in all conditions tested with modest or no induction of *aprA/mcr7* expression at acidic pH, high zinc concentration, or in macrophages; (ii) on the contrary, PhoPR-STB-J is associated with a strong constitutive expression of *aprA*/*mcr7;* (iii) between these two extremes, PhoPR-HN878 and PhoPR-STB-D provide conditional expression of *aprA*/*mcr7* in acidic, high zinc conditions and during intracellular growth; (iv) finally, PhoPR-bovis-B is not activated at acidic pH and high zinc concentration *in vitro* but provides the strongest expression of *aprA*′::GFP during macrophage infection.

### Expression of *phoPR* variants in HN878 modulates the pathogenicity in the mouse model

**Fig 7:**
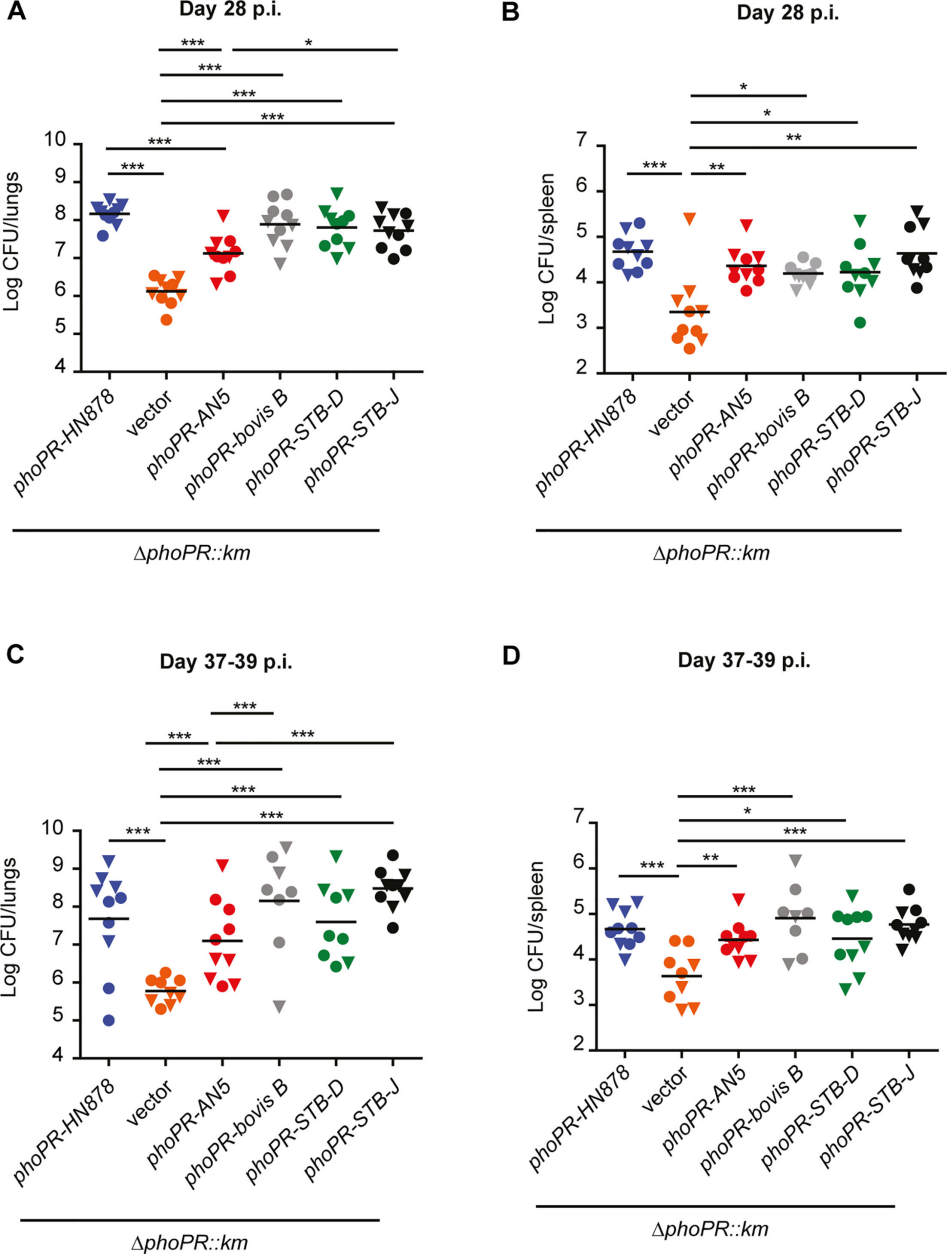
*phoPR* variants in HN878 impact the bacterial loads in the lungs and spleen of infected mice. Six groups of ten 6- to 7-week-old female C3HeB/FeJ mice were intranasally infected with approximately 100 colony-forming units (CFUs) of recombinant strains. In two independent experiments, pulmonary and splenic bacterial loads were quantified at 28 days post-infection (p.i.) (**A and B**) and at 37–39 days p.i. (**C and D**). Each symbol represents the bacterial burden in a single organ from an individual mouse; triangles and circles correspond to mice from experiment 1 and experiment 2, respectively. Estimated marginal means (emmeans) were calculated using a *post hoc* test to assess differences between groups, following a linear mixed-effects model in which experiments A and B were included as random effects to account for potential batch effects and organs were included as fixed effects. Asterisks indicate BH-adjusted *P*-values for emmeans-based group comparisons. *, *P* < 0.05; **, *P* < 0.01; ***, *P* < 0.001.

Finally, we aim to correlate PhoPR activity with the pathogenicity of the recombinant strains in cellular and animal models. First, we evaluated the virulence of HN878 WT, the Δ*phoPR* mutant, and strains expressing the *phoPR* variants in infected hMDAMs by quantifying the percentage of infected cells and the number of bacteria per cell. Comparative analysis indicated that the expression of *phoPR* variants in HN878 background did not affect bacterial infectivity in hMDAMs for up to 5 days post-infection (p.i.) (Fig. S8). Second, six groups of C3HeB/FeJ mice were infected intranasally with approximately 100 colony-forming units (CFU), and we evaluated the bacterial load in lungs and spleen at 28 days and 37 or 39 days p.i. (Fig. 7). As expected, we found a significant reduction in the bacterial load for the HN878 Δ*phoPR::km* mutant strain in comparison to the four complemented strains tested, at both time points and in both organs (Fig. 7 and Fig. S9). These results indicate that the expression of all the *phoPR* variants in the HN878 Δ*phoPR::km* mutant enhances the bacterial capacity to survive/multiply in mice in comparison to the *phoPR* knock-out strain. Among the complemented strains, the bacterial loads were quite similar in the spleen both at day 28 and days 37/39 p.i. (Fig. 7B and D and Fig. S9). In contrast, on day 28, the mice infected with the strain expressing *phoPR-AN5* displayed significantly lower bacillary charge in the lungs than the mice infected with the strains expressing *phoPR-HN878* and *phoPR-STB-J* (Fig. 7A and Fig. S9). On days 37/39, the CFU values in the lungs were more dispersed, as previously observed by Irwin and collaborators (37), and, although the mice infected with the strain expressing *phoPR-AN5* displayed lower bacillary charge, the reduction in the bacterial load was statistically significant only when compared with the *phoPR-STB-J-* and *phoPR-bovis-B-*complemented strains (Fig. 7C).

**Fig 8:**
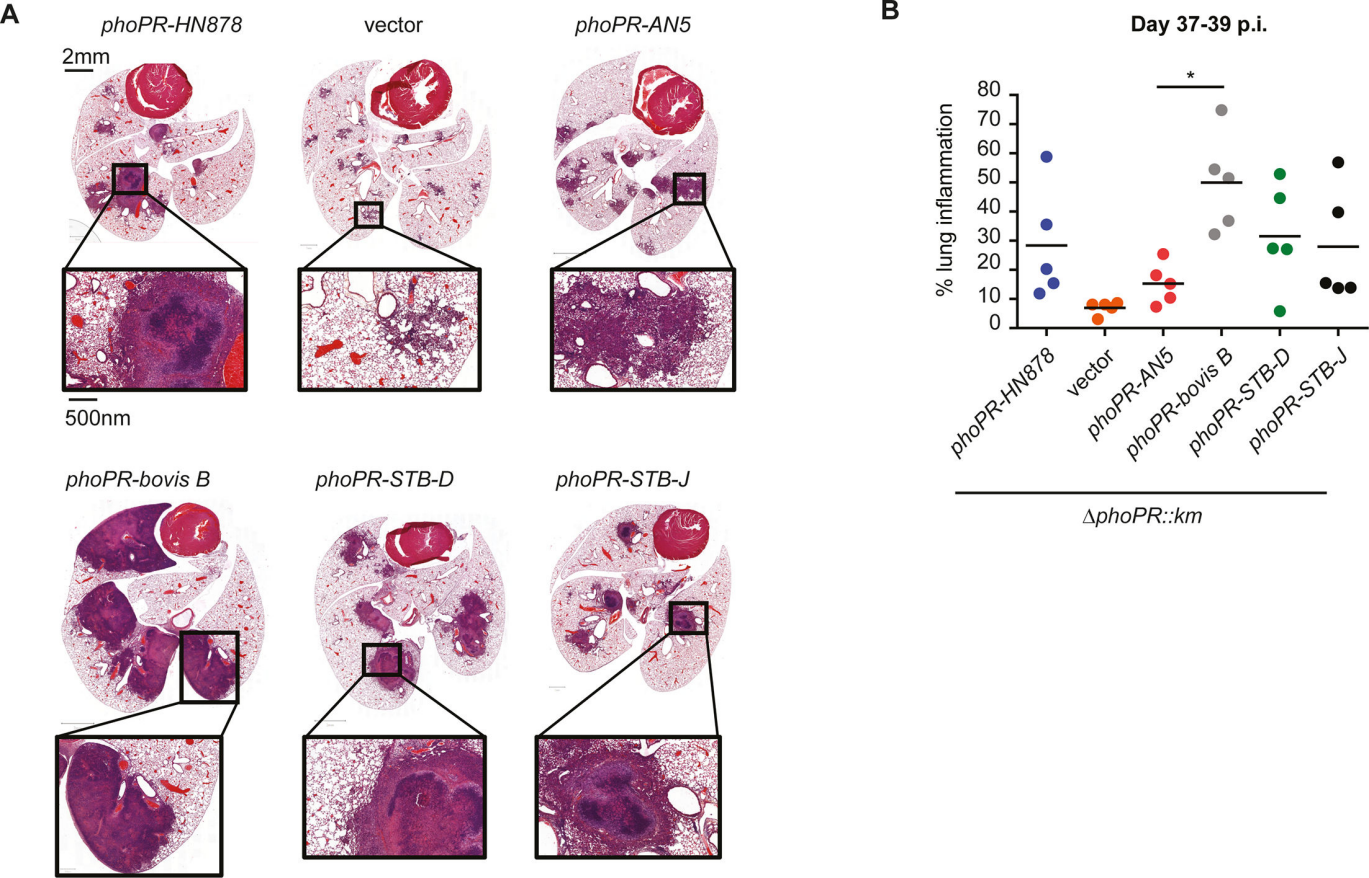
*phoPR* variants in HN878 differentially modulate lung inflammation in C3HeB/FeJ mice. C3HeB/FeJ mice were intranasally infected with six different recombinant *M. tuberculosis* HN878 strains carrying distinct *phoPR* variants (*n* = 5 mice per strain; total *n* = 30). Pulmonary inflammation was assessed at 37–39 days post-infection. Lung tissues were formalin-fixed, paraffin-embedded, and stained with hematoxylin and eosin (H&E) to visualize histopathological lesions (**A**). The inflamed lung area was quantified as a percentage of the total lung surface using QuPath software based on manual delineation of inflamed regions (**B**). Each data point represents an individual mouse. This experiment was performed once. *, *P* < 0.05.

Histopathological analysis showed that, after 28 days of infection, there is a trend for a lower percentage of inflamed areas in the lungs of mice infected with the Δ*phoPR::km* mutant and strains expressing the *phoPR-AN5* and *phoPR-bovis-B* variants (area of lung infiltration <10% on average) than the others (Fig. S10). A higher percentage of inflamed areas (area of lung infiltration >10% on average) was observed for the lungs of mice infected with strains expressing the *phoPR-HN878, phoPR-STB-D,* and *phoPR-STB-J* variants, with greater intermouse heterogeneity (Fig. S10). After 37 days of infection, the area of pulmonary inflammation increased, except in mice infected with the Δ*phoPR::km* mutant (Fig. 8). However, this increase was modest for the mice infected with the *phoPR-AN5* recombinant strain in comparison to the *phoPR-HN878*, *phoPR-STB-D*, and *phoPR-STB-J* groups, for which the lung inflammation reached an area around 30%, again with significant intermouse heterogeneity (Fig. 8). Remarkably, mice infected with the *phoPR-bovis-B* recombinant strain showed pulmonary inflammation with extensive lung lesions, which was significantly higher than those of *phoPR-AN5* (Fig. 8).

Altogether, our results demonstrated that the expression of *phoPR-AN5* in the HN878 background provides lower virulence than the expression of the other three variants.

## DISCUSSION

The PhoPR system is among the most extensively studied regulatory system in *MTB*, revealing it to be a key regulatory system that controls multiple functions essential for pathogenicity. Recent findings have established that the *phoR* gene, coding for the sensor kinase, is under strong positive selection, accumulating nonsynonymous mutations since the emergence of MTBC and *M. canettii* bacilli (8, 10). However, the consequences of these mutations on their adaptation to the human host remain poorly documented. Our research identifies zinc (Zn²^+^) and cadmium (Cd²^+^) as novel extracellular inducers of the PhoPR system in *MTB* in addition to acidic pH, and we find that lineage-specific mutations in animal-adapted strains and *M. africanum* (L5 and L6) or in *M. canettii* STB-J impairs the activation of the PhoPR system by these signals.

The finding that Zn²^+^ is an activator of the PhoPR system is relevant for the biology of *MTB* because the pathogen faces bursts of free zinc upon colonization of macrophages, with the concentration of this cation increasing up to ~450 µM in the *MTB* phagosome (31). The exact mechanism through which Zn²^+^ and Cd²^+^ activate the sensor protein PhoR remains undefined. We hypothesized that these divalent cations may directly bind to PhoR’s sensor domain, potentially causing a conformational shift that activates PhoR. This mechanism is analogous to that of PhoQ in *Salmonella enterica*, which binds divalent cations (Mg²^+^, Ca²^+^, and Mn²^+^) via a negatively charged “acidic patch” located in its periplasmic domain (38–40). Such binding creates a metal bridge between the PhoQ protein and the negatively charged plasma membrane, maintaining the protein in a repressed state (39, 41). Using the predicted structural models of PhoR, acidic residues in the sensor domain were identified as possible binding sites for Zn²^+^ and Cd²^+^ (Fig. S11). However, Zn²^+^ and Cd²^+^ activate PhoPR in *MTB* rather than repressing it, as in *S. enterica* PhoQ. In addition, the distance of the PhoR “acidic patch” from the plasma membrane might preclude metallic bridging. Therefore, if this hypothesis is true, the exact mechanism of PhoP activation by Zn²^+^ and Cd²^+^ remains to be established, as is the case for activation by acidic pH. An alternative hypothesis would be an indirect activation pathway, potentially involving β-carbonic anhydrases (42–44). Carbonic anhydrases are metalloenzymes that incorporate Zn²^+^ to catalyze the hydration of CO₂ to bicarbonate (HCO₃^−^) and protons (H^+^) and to maintain pH homeostasis (45). Therefore, increasing Zn²^+^ concentration may enhance carbonic anhydrase catalytic activity, lower periplasmic pH, and thereby induce PhoR activation. Of note, we did not observe synergistic effects between Zn²^+^, Cd²^+^, and acidic pH in PhoPR activation. These results suggest either a shared activation mechanism, for instance, *via* the same site in PhoR, or maximal activation by independent signals. Resolving the structure of the full-length PhoR or of its sensor domain could help clarify this activation mechanism.

**Fig 9:**
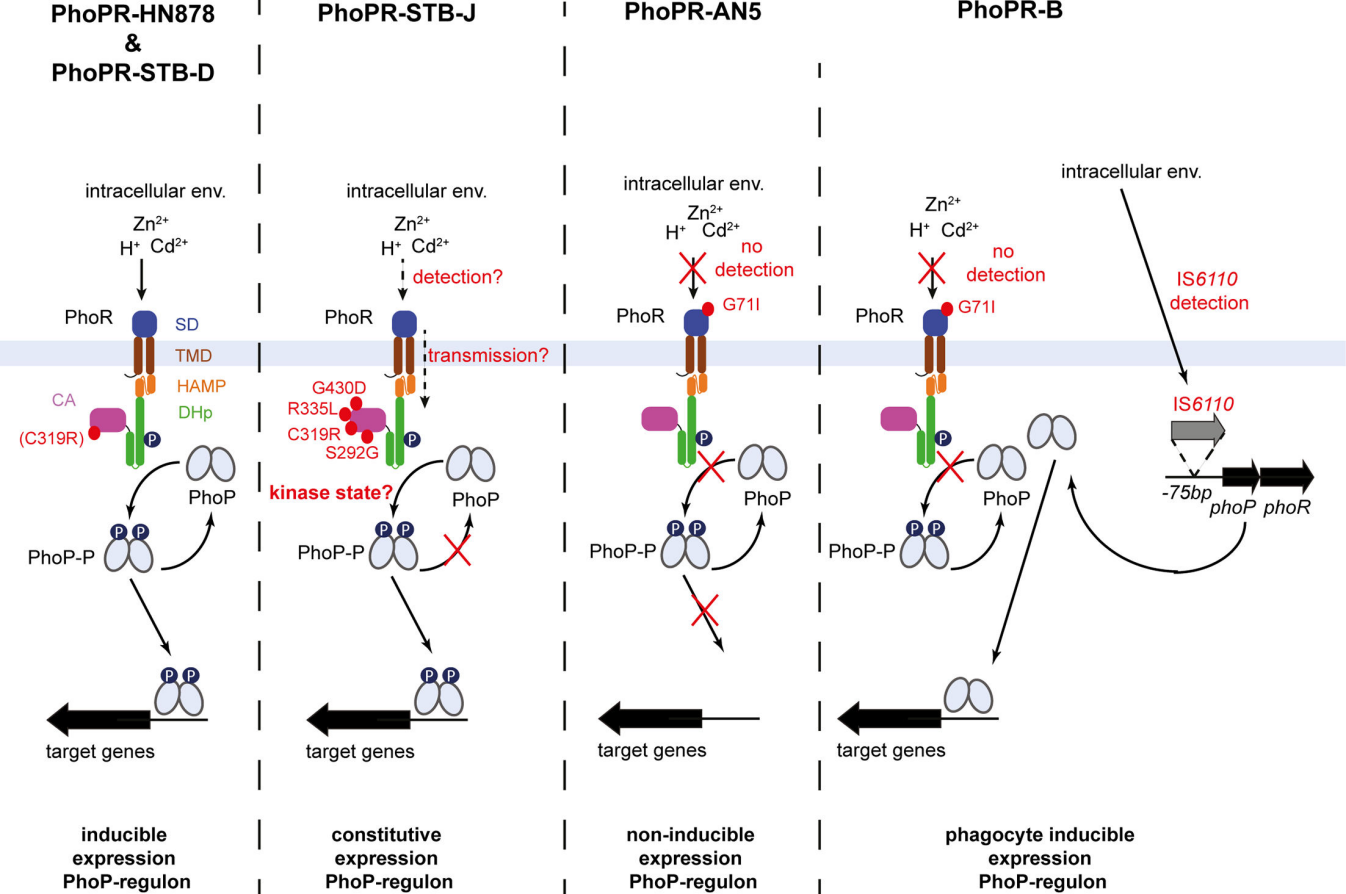
PhoPR variants and signal recognition. The PhoPR systems from *MTB* HN878 and *M. canettii* STB-D (with the PhoR C319R mutation for PhoR-STB-D) respond to acidic pH, excess zinc, and the intramacrophage environment. Signal detection is followed by transduction through the functional domains of PhoR, resulting in phosphorylation of the response regulator PhoP and subsequent activation of the PhoP regulon. The PhoPR-HN878 variant also responds to cadmium exposure (PhoP-STB-D variant was not tested with cadmium). The PhoR protein of *M. canettii* STB-J exhibits constitutive activity under all tested environmental conditions, likely due to a permanently active kinase state probably conferred by four mutations within its catalytic ATP-binding domain. However, its ability to sense and transduce environmental signals remains unconfirmed. The G71I mutation in the sensor domain of PhoR in *M. bovis* AN5 impairs signal detection, resulting in a lack of PhoP regulon activation. The PhoPR system from *M. bovis* B is unresponsive to acidic pH and zinc but remains active within macrophages. This activity is potentially driven by an IS6110 insertion upstream of *phoP*, which may enhance *phoP* expression or indirectly influence PhoP activation. Domain annotations: SD, sensor domain; TMD, transmembrane domain; HAMP, signal relay domain; DHp, dimerization and histidine phosphotransfer domain; CA, catalytic ATP-binding domain; P, phosphoryl group.

In this study, we confirm that several lineage- or strain-specific mutations identified within the *phoPR* are not neutral. Our results suggest that these mutations impact the functioning of the PhoPR system, specifically its ability to detect and respond to signals for activating the PhoP regulon. We identified four distinct behaviors of *phoPR* variants in response to acidic pH, zinc excess, and the intramacrophage environment (Fig. 9). First, PhoPR-HN878 responds to acidic pH, high Zn^2+^ concentration, and the intramacrophage environment. Unexpectedly, PhoPR-STB-D exhibits the same behavior as PhoPR-HN878. This result indicates that the C319R mutation near the catalytic site of PhoR-STB-D does not affect the signal response. In a previous study, we found that PhoPR-regulated functions, such as EsxA secretion and Ac_4_SGL production, are less expressed in STB-D than in HN878 under standard growth conditions (27). Our latest findings suggest that the low expression of the PhoP regulon in STB-D is not due to a defect in PhoPR. One hypothesis is that the basal activation level of PhoPR is higher in HN878, and potentially in *MTB* strains in general, than in *M. canettii* STB-D. A recent study showed that PhoP interacts with the PrrB protein, a sensor kinase of another TCS in *MTB* (35). A differential activity of PrrB or carbonic anhydrases between *MTB* and *M. canettii* could account for the differences in PhoP-regulated function expression between these two bacteria. Further studies are required to clarify this point. Second, consistent with the findings of our previous work (26), we confirm that the PhoR G71I mutation affects the sensor’s activity. Here, we show that this mutation reduces the PhoR response to acidic pH and Zn^2+^ in *MTB*. This reduced induction of the PhoP regulon by different signals is observed in PhoPR-AN5 and PhoPR-bovis-B, both of which carry the PhoR G71I mutation. However, the expression of genes in the PhoP regulon is higher in the strain expressing *phoPR-AN5* or *phoPR-bovis-B* compared to the Δ*phoPR::km* mutant, indicating some PhoPR activity. These results are consistent with those of other studies on *M. bovis* (46, 47), which demonstrate that the inactivation of *phoP* or *phoPR* affects the expression of genes in the PhoP regulon within this phylogenetic lineage and impacts growth under acidic conditions. The weak activation of the PhoP regulon under acidic pH conditions or in the presence of Zn^2+^ indicates that the PhoPR-AN5 and PhoPR-bovis-B are less potent than PhoPR-HN878 to respond to these environmental cues, at least in *MTB*. However, the presence of IS*6110* upstream of *phoPR* in *phoPR-bovis-B* enables PhoP-regulon activation when bacteria are in the macrophage environment, a phenomenon not observed with *phoPR-AN5*, and thus defining the third behavior. This result aligns with the presence of a promoter in IS*6110* that is activated in phagocytes, enhancing the expression of downstream genes (12, 48). Finally, the fourth behavior is that of PhoPR-STB-J, which is associated with constitutive activation of the PhoP regulon and is insensitive to pH, Zn^2+^, or macrophage infection. The PhoR-STB-J protein contains four mutations in the catalytic domain (Fig. 1). Given their localization, it is likely that these mutations induce conformational changes in the PhoR protein, maintaining it in a kinase-active state. Specifically, the aspartic residue substitution G430D may potentially interact with two other arginine residues at positions 296 and 420, stabilizing the structure (27).

Regarding the impact of different PhoPR variants on the pathogenesis of *MTB* in a murine model, our results show that the expression of all variants significantly increases the bacterial load in the lungs and spleen at both time points analyzed in comparison to the Δ*phoPR::km* strain. These results confirm that all variants retain some level of activity, in accordance with previous findings (26, 27, 35, 46). However, we observe that higher activity of the PhoPR system increases the virulence of *MTB*. This is reflected in a lower bacterial and lesion load in the lungs of mice infected with the strain expressing PhoPR-AN5 compared to those infected with HN878-derived strains expressing the other variants. Surprisingly, the strain expressing PhoPR-bovis B induces the strongest pulmonary inflammation, with a dramatic increase between time points J28 and J37-39. These findings support our previous results suggesting that the insertion of IS*6110* upstream of *phoPR* compensates for the loss of activity associated with the G71I mutation in PhoR. This compensation is likely linked to the activation of the internal IS*6110* promoter in response to the intracellular environment of macrophages. Maintaining the ability to multiply and survive in the host is a prerequisite for a successful infectious cycle of TB bacilli. Recent studies show that IL-1R-dependent early migration of infected alveolar macrophages to lymph nodes is as important as bacterial load for the induction of lung lesions, such as cavities, favoring release of bacteria in the airways and possibly transmission (49). This early migration requires EsxA secretion, which depends on PhoPR (22, 50). Therefore, it remains to be explored whether PhoPR variants confer *MTB* with similar abilities to initiate early alveolar dissemination to produce granulomas with the potential to develop into cavities rather than general lung inflammation that can be lethal for the host but not very effective for transmission.

In conclusion, our results provide new insights into the impact of mutations in *phoPR* on the biology and evolutionary history of MTBC and *M. canettii* bacilli. They also pave the way for analyzing the mechanisms of PhoR activation by identifying mutations that either maintain PhoR in an active conformation or block its activation by various signals.

## MATERIALS AND METHODS

### Bacterial strains, plasmids, and culture conditions

The strains and plasmids are described in Table S1. These strains were cultured in Middlebrook 7H9 liquid medium (Difco) containing ADC (0.2% dextrose, 0.5% BSA fraction V, and 0.0003% beef catalase) and 0.05% Tween 80 when indicated and on solid Middlebrook 7H11 broth containing OADC (0.005% oleic acid, 0.2% dextrose, 0.5% BSA fraction V, and 0.0003% beef catalase). When required, kanamycin (40 µg/mL), streptomycin (40 µg/mL), and hygromycin (50 µg/mL) were added to the culture medium.

When indicated, NaCl (500 mM; EUROMEDEX) or metal ion ZnSO_4_, CuSO_4_, MgSO_4_, or CdSO_4_ (500 µM; Sigma), DETA/NO (5 mM; Sigma), or H_2_O_2_ (10 mM; Sigma) was added to the medium. Finally, when necessary, 100 mM MES buffer (Sigma) was used to adjust and maintain an acidic pH of 5.7 or 4.5. For media at pH 4.5, Tween 80 was replaced by tyloxapol (0.05% final), and the medium was also supplemented with a carbon source, i.e., 0.2% glycerol or 200 µM oleic acid (OA), to enable bacterial growth (51). For cultures under hypoxic conditions, bacteria were placed in a hypoxia station set to 2% O_2_ or 15% O_2_.

### Recombinant strain and plasmid constructions

Construction of the ∆*phoPR ::km* mutant (also named PMM335) in the *MTB* HN878 strain was carried out using the recombinant allelic exchange technique (Fig. S1) (52).

Recombinant strains were obtained by complementation of the Δ*phoPR::km* mutant (PMM335) with the *phoPR* variants of *MTB* strains HN878, *M. canettii* (STB-D and STB-J), and *M. bovis* (AN5 and B). These variants were amplified by PCR from genomic DNA (with the exception of *phoPR-bovis-B*) using primers EM28 + EM30, EM28 + EM31, EM29 + EM32, or EM29 + EM23 and inserted within the PmeI restriction site in plasmid pEM08 (Table S1), which was generated by ligation between the OriE, att, integrase region from plasmid pMV361 (Addgene), and a streptomycin resistance cassette. The resulting plasmids were named pEM09 (*phoPR-HN878*), pEM10 (*phoPR-AN5*), pEM11 (*phoPR-STB-D*), and pWM424 (*phoPR-STB-J*). The plasmid pEM14 carrying *phoPR-bovis-B* was generated by inserting the *phoPR* gene obtained by PCR amplification with primer EM28+EM33 from *M. bovis* AN5 genomic DNA to generate plasmid pEM13, followed by insertion of a 2-kb AgeI fragment from pSO7 plasmid (12) into the AgeI site of pEM13. The sequence of each *phoPR* variant was confirmed by Sanger sequencing. The control vector for complementation was constructed by insertion of a complementation fragment deleted from PhoP and the 5′ part of PhoR obtained by PCR amplification using primers EM34 + EM35 and HN878 genomic DNA into plasmid pEM08.

The fluorescent reporter plasmid pEM12 was constructed by cloning the *aprA'*::GFP, *smyc':*:mCherry region of the replicative plasmid pMT-3 (16), obtained by PCR amplification using primers EM23+orim, between the NheI-HindIII restriction sites of the integrative plasmid pMV366H (Addgene).

### Western blot analysis

Immunoblot analysis of secreted proteins (5 µg) was performed as previously described (27) using primary antibodies (anti-EsxA [Abcam] and anti-Apa [BEI Resources]) and secondary HRP-conjugated goat anti-mouse antibodies (BioRad) at dilution 1:1,000 and 1:5,000, respectively.

### Lipid analysis

Crude lipid extracts were obtained from 100 mL culture grown for 8 weeks in 7H9 ADC, as previously described (27, 30).

### RNA extraction and RT-qPCR analyses

Mycobacterial RNAs were extracted from cultures with OD 600 nm between 0.5 and 0.8. The RNeasy Kit (Qiagen) was used, with a few modifications to the protocol. Cultures (7 mL) were centrifuged for 10 min at 4,000 rpm. The pellets were washed with 1 mL PBS (Gibco) and centrifuged again for 10 min at 4,000 rpm. The pellets were then transferred to 750 µL of RLT lysis buffer containing 0.1% β-mercaptoethanol (Sigma). Bacteria were lysed with glass beads (0.1 µm diameter) added to the buffer. Tubes were shaken with a bead-beater for 2 min at maximum power. After centrifugation for 2 min at 14,000 rpm, the supernatants were recovered. This lysis step was performed a second time. Bacterial lysates were then double-filtered on 0.2-µm PES filters (Fisher Scientific) before adding an equivalent volume of absolute ethanol. RNAs were extracted according to the procedure mentioned in the kit.

RNAs were then treated with DNaseI (Fisher Scientific) followed by a reverse transcriptase (Fisher Scientific, Invitrogen SuperScript III First-Strand Synthesis System) to obtain cDNAs.

Real-time PCRs were performed with a DNA intercalant (Ozyme, SensiFAST SYBR Hi-ROX Kit) following the manufacturer’s recommendations. An ABI Prism 7500 (7500 Real-Time PCR System, Applied Biosystems) was used for real-time PCRs, and data were analyzed with 7500 v2.0.6 software (Applied Biosystems). The *sigA* gene was used as a housekeeping gene. ΔCt (cycle threshold) values were plotted directly, or the 2^-ΔΔCt^ method was used to calculate the fold change in different conditions.

### Production of human monocyte-derived alveolar macrophages and infection

Human blood of anonymous nontuberculous donors was supplied by the Etablissement Français du Sang de Toulouse. Peripheral blood mononuclear cells (PBMCs) were isolated by Ficoll gradient centrifugation (EUROBIO) as previously described (53). Monocytes were then purified from other cells on a column using micromagnetic beads coupled to anti-CD14 antibodies (Miltenyi Biotec) and allowed to adhere to 24-well plates containing a glass coverslip. The medium was then supplemented to a final concentration of 10% heat-inactivated fetal bovine serum (FBS, Gibco) and 10 ng/mL GM-CSF (Preprotech) to allow their differentiation into GM-CSF monocyte-derived macrophage with a phenotype close to that of alveolar macrophages (hMDAMs) (54).

The mycobacterial infection was done as previously described (55). To visualize macrophages, cells were incubated with 15 µM CellTracker Violet BMQC (Molecular Probes) at 37°C in 5% CO_2_ for 30 min. Cells were then washed and fixed with 4% paraformaldehyde (PFA) for 2 h.

The coverslips were mounted on glass slides using Dako Fluorescence Mounting Medium (Dako) and analyzed by confocal microscopy.

### Confocal microscopy analyses

Z-scan images of the samples were acquired using a Zeiss LSM710 two-photon confocal microscope (×63 immersion objective). Confocal images were reconstructed in 3D and analyzed using Imaris software. Macrophages are visualized in blue using violet BMQC signal, and bacteria are visible in red as they constitutively produce mCherry. Quantification of the GFP reporter signal produced by bacteria was done, as described by reference 15.

### Flow cytometry analysis

To measure bacterial GFP and mCherry fluorescence, we used an LSRFortessaTM flow cytometer. Briefly, 1 mL of the bacterial culture was fixed with 4% PFA for 2 h in the dark, and then the bacteria were centrifuged at 12,000 rpm and PFA removed. Bacteria were washed with 1 mL PBS and centrifuged again at 12,000 rpm, before being resuspended with 500 µL of this solution. Where necessary, the bacteria were diluted in a larger volume of PBS to ensure that no more than 10,000 events per second were recorded during aspiration of the liquid by the cytometer. Data were analyzed using FlowJo software (FlowJo LLC, version 10.2.6) (Fig. S12).

### Virulence studies in mice

C3HeB/FeJ mice, 7 to 8 weeks old, were infected intranasally with approximately 10^2^ CFU of the indicated strains. C3HeB/FeJ mice were euthanized at 28 days and 37–39 days p.i., and either bacterial load was evaluated in the lungs and spleen by plating organ homogenates on 7H11 OADC or the lungs were processed for histopathological analyses. In those cases, the lungs were fixed for 48 h in 10% neutral buffer formalin before transferring them into 70% ethanol. After the lungs were embedded in paraffin, tissue samples were sectioned (5 µM) and stained with hematoxylin and eosin. Histomorphological scoring of TB lesions was determined by measuring the area of the infiltrated lung tissue using the Qupath software (56).

### Statistical analysis

Statistical analyses and data visualization were carried out using either GraphPad Prism or the R working environment (57), under the RStudio interface, and the following packages: lme4, emmeans, multcomp, tidyverse, and ggtext (58–60). For experiments on hMDAMs treated or not with bafilomycin, the significant difference between individual groups was determined by one-way variance analyses followed by Bonferroni’s multiple comparison test. *P* values lower than 0.05 were considered statistically significant. For the experiments with recombinant strains expressing *phoPR* variants, differences in log-transformed bacterial burden values between groups were determined by estimated marginal means (emmeans) following the implementation of mixed linear models. In the case of mice bacterial burden, independent experiments were included as random intercepts in the model to account for potential batch effects, and strains and organs were included as fixed effects. In hMDAM studies, donors were implemented as random intercepts and strains and dpi as fixed effects. Benjamini-Hochberg-adjusted *P* values lower than 0.05 were considered statistically significant (61). R scripts are available upon request.

## SUPPLEMENTAL MATERIAL

Supplemental material mbio.00939-26-s0001.pdf Supplemental information, Figures S1 to S12, and Tables S1 and S2.

ASM does not own the copyrights to Supplemental Material that may be linked to, or accessed through, an article. The authors have granted ASM a non-exclusive, world-wide license to publish the Supplemental Material files. Please contact the corresponding author directly for reuse.
