# Supplementary material for: PhoPR variants from several phylogenetic lineages of tuberculosis bacilli respond differently to extracellular signals
Source: mBio. 2026 May 29;17(7):e00939-26. doi: 10.1128/mbio.00939-26 (PMC13343901; doi:10.1128/mbio.00939-26)
Supplement: Supplemental material — Supplemental information, Figures S1 to S12, and Tables S1 and S2. [file mbio.00939-26-s0001.pdf]

PhoPR variants from several phylogenetic lineages of tuberculosis bacilli respond differently to extracellular signals

Authors: Eva Meunier*[1,2], Carlos Adriano de Matos e Silva*[1], Wladimir Malaga[1], David Rengel[1], Lionel Mourey[1], Catherine Astarie-Dequeker [1#], Christophe Guilhot [1#]

[1] Institut de Pharmacologie et de Biologie Structurale (IPBS), Université de Toulouse, CNRS, UPS, Toulouse, France

[#] Address correspondence to Catherine Astarie-Dequeker, Catherine.Astarie-Dequeker@ipbs.fr or Christophe Guilhot, Christophe.Guilhot@ipbs.fr

* These authors contributed equally to this work.

[2] Present address: Institut Pasteur de Nouvelle-Calédonie, Nouméa, Nouvelle-Calédonie.

Running title: PhoPR variants in tuberculosis bacilli

This file include:

- Supplementary information
- Supplementary figures and legends
- Supplementary table

Supplementary information

Construction of a set of recombinant strains derived from *Mycobacterium tuberculosis* HN878

To explore the impact of SNPs in *phoPR* on response to signals, we selected several PhoPR variants associated with strains displaying various expression of PhoPR controlled functions. As a reference, we chose the PhoPR variant of *M. tuberculosis* HN878 (PhoPR-HN878), a strain efficiently transmitted in humans, because the *phoPR-HN878* sequence is a good representative of that in *MTB*. For instance, the PhoP-HN878 and PhoR-HN878 amino acid sequences are identical to that of PhoP and PhoR from several reference *M. tuberculosis* strains such as CDC1551 or Erdman. This is not the case with the H37Rv strain, classically used in research laboratories, which has a specific mutation leading to a proline in position 172 instead of a leucine (Leu172Pro). This mutation is located in the transmembrane domain of the PhoR protein and modifies the response to acid stress and the PhoP-regulon expression (1, 2).

The PhoPR-AN5 and the PhoPR-bovis-B, when compared with PhoPR-HN878, have a mutation Gly71Ile located in the periplasmic sensor domain of PhoR (3) (Figure 1). This mutation is not strain-specific, as it is also found in all animal-adapted strains and strains of the *M. africanum* groups (3). We previously showed that, the PhoP regulon of *M. bovis* is under-expressed and production of the lipids DAT/PAT, SL is greatly diminished *in vitro* (3). *M. bovis B* strain, but not AN5 and most *M. bovis* strains, also has the insertion sequence IS*6110* within the promoter region of the *phoPR* genes, which increases PhoP regulon expression and DAT/PAT and SL synthesis (3, 4). Regarding the transmission, most *M. bovis* strains, unlike HN878, do not transmit efficiently in humans, while *M. bovis B* triggered a chain of human-to-human transmission (5). Finally, we selected the last two PhoPR variants (PhoPR-STB-D and PhoPR-STB-J) from strains, STB-D and STB-J, in the *M. canettii* group because these strains have not been associated with transmission between humans. The STB-D strain displays the most common PhoPR variant among *M. canettii* strains (1). It has a cysteine instead of an arginine at position 319 (Arg319Cys) in the catalytic domain of PhoR in comparison to PhoPR-HN878 (Figure 1). Expression of PhoP-controlled functions in STB-D are lower than in *MTB*, as in most of the other *M. canettii* strains studied strains (1). The PhoPR-STB-J variant displays four substitutions in the catalytic domain of PhoR in comparison to PhoR-HN878: Arg319Cys, Ser292Gly, Arg335Leu and Gly430Asp (Figure 1). This highly active variant leads to higher expression of PhoP-regulated genes and the production and secretion of greater quantities of Ac_4SGL (tetraacylated sulfoglycolipid, the major form of SL) and EsxA than the STB-D strain. However, the virulence of STB-D and STB-J is similar in mice (6) and no transmission was ever described in humans (7, 8). For each construct, the upstream region including the *phoPR* promotor was included in the complementation plasmid, so the *phoPR* genes are expressed from their native promotor. It is important to mention that all the recombinant strains generated in our work produce the same PhoP protein. Therefore, all the results obtained in our study are solely due either to the mutations present in *phoR* gene or to the IS*6110* insertion upstream *phoPR*.

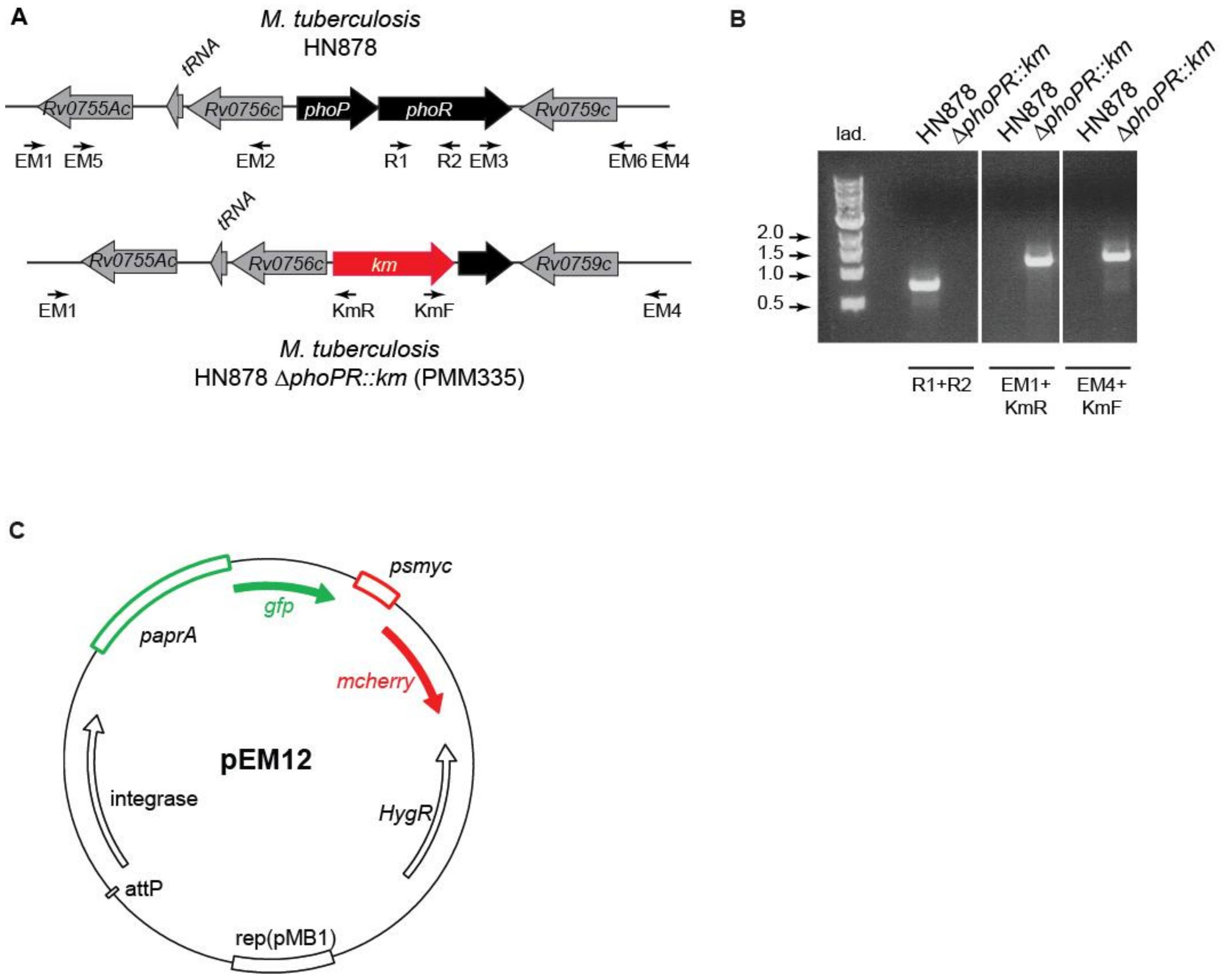

Supplementary Figure 1: Construction of HN878 Δ*phoPR::km* mutant strain, map of the fluorescent-reporter plasmid.

(A) Schematic representation of the *phoPR* locus in *Mycobacterium tuberculosis* HN878 and in the HN878 Δ*phoPR*::*km* mutant, in which the native *phoPR* operon has been replaced by a kanamycin resistance gene (*Km*). Briefly, for the mutant construction, three PCR fragments were amplified and then fused to form an allelic exchange substrate (AES). Two 1 kb fragments, upstream of *phoP* and downstream of *phoR*, were generated from HN878 genomic DNA by PCR amplification with PrimeSTAR polymerase (TAKARA) using primers EM1+EM2 and EM3+EM4 (Supplementary Table 1 and Supplementary Figure 1). The third fragment containing a kanamycin resistance cassette (*km*) was amplified using primers EM7+ EM8 from the pET28a vector (addgene). The three PCR fragments were purified with the QIAquick®PCR Purification Kit (Qiagen) and fused together by PCR with Prime STAR polymerase to obtain a single 3 kb fragment. The AES was cloned at the EcoRV site into plasmid pJET1.2/blunt (ThermoFisher Scientific) to give plasmid pEM03.

The AES was amplified using primers EM5/EM6, purified and then transformed by electroporation into the recombinant strain HN878 expressing the recombination system from plasmid pJV53H (9).
Primers EM1 to EM6, used for PCR verification of the mutant genotype, are indicated on the diagram.
Transformants selected on 7H11 medium containing kanamycin were analyzed by PCR using primer pairs R1/R2, EM1/KmR and EM2/KmF. A clone with the expected PCR profile was selected and named Δ*phoPR* ::*km* (or PMM335).

(B) PCR analyses of the *M. tuberculosis* HN878 Δ*phoPR* ::*km*

(C) Map of the reporter plasmid pEM12. This construct was generated by subcloning the dual reporter cassette *paprA'::gfp, psmyc'::mcherry* from pMT-3 (10) into the integrative vector pMV366H (Addgene). pEM12 includes a mycobacteriophage Ms6-derived integration system composed of the *attP* site and the gene encoding the integrase, allowing stable chromosomal integration in mycobacteria. The plasmid also contains a hygromycin resistance marker (*HygR*) for selection in both *E. coli* and mycobacteria. Expression of the red fluorescent protein mCherry is driven by the constitutive *psmyc* promoter, whereas GFP expression is regulated by the PhoPR two-component system via the *paprA* promoter. This system allows the integration of the *aprA'::GFP, smyc'::mCherry* reporter system onto the chromosome enhancing the reporter system stability (data not shown) and avoiding confounding effect associated with a multicopy plasmid.

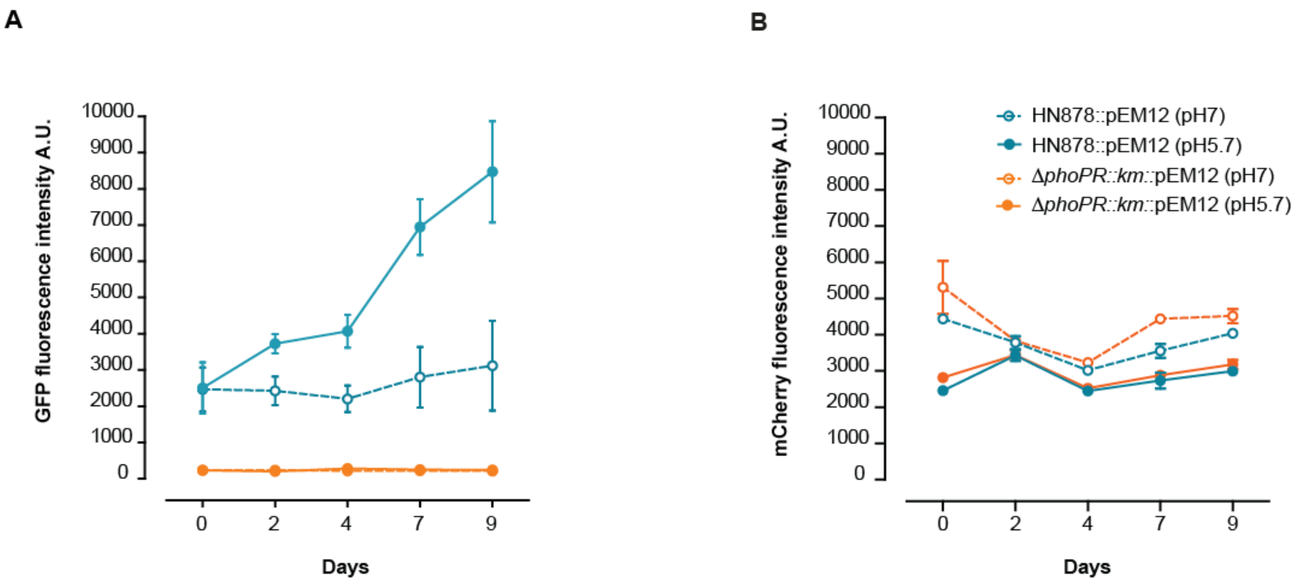

Supplementary Figure 2: GFP fluorescence at neutral or acidic pH in recombinant HN878 strains expressing the fluorescent reporter.

Inducible GFP fluorescence **(A)** and constitutive mCherry fluorescence **(B)** were measured in *M. tuberculosis* HN878 harboring the pEM12 reporter plasmid and cultured at different time points under neutral (pH 7) or acidic (pH 5.7) conditions. Values represent the mean fluorescence intensity (MFI) measured by flow cytometry from a gated population of 50,000 recorded events. Error bars represent the mean ± standard deviation of three independent experiments (n = 3).

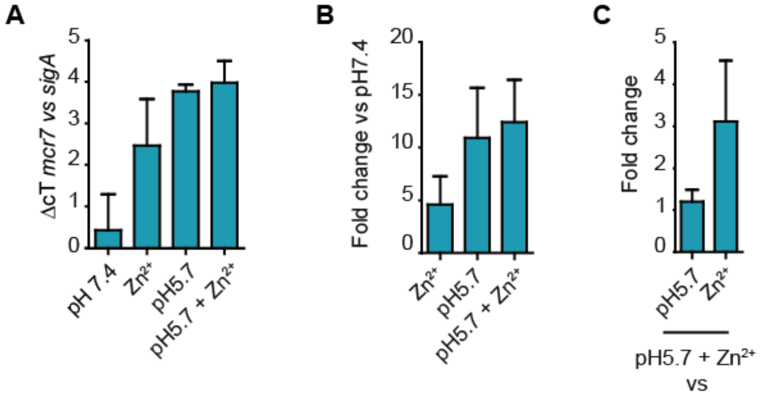

Supplementary Figure 3: Zinc and acidic pH do not induce synergistic effect on PhoPR activity.

Relative expression of the *mcr7* gene, normalized to the housekeeping gene *sigA*, in WT HN878 after exposure to acidic pH (pH 5.7), zinc stress ($ZnSO_4$, 500 µM), or combined Zn^{2+}+acidic pH (Panel A). The induction of *mcr7* under stress conditions is shown as fold change relative to the baseline expression observed at pH 7.4 (Panel B) or as fold change of expression after Zn^{2+}+acidic pH exposure relative to the expression observed at pH 5.7 or after zinc stress (Panel C). Gene expression was quantified by RT-qPCR. Error bars represent the mean ± standard deviation from two independent experiments (n = 3) performed in triplicate.

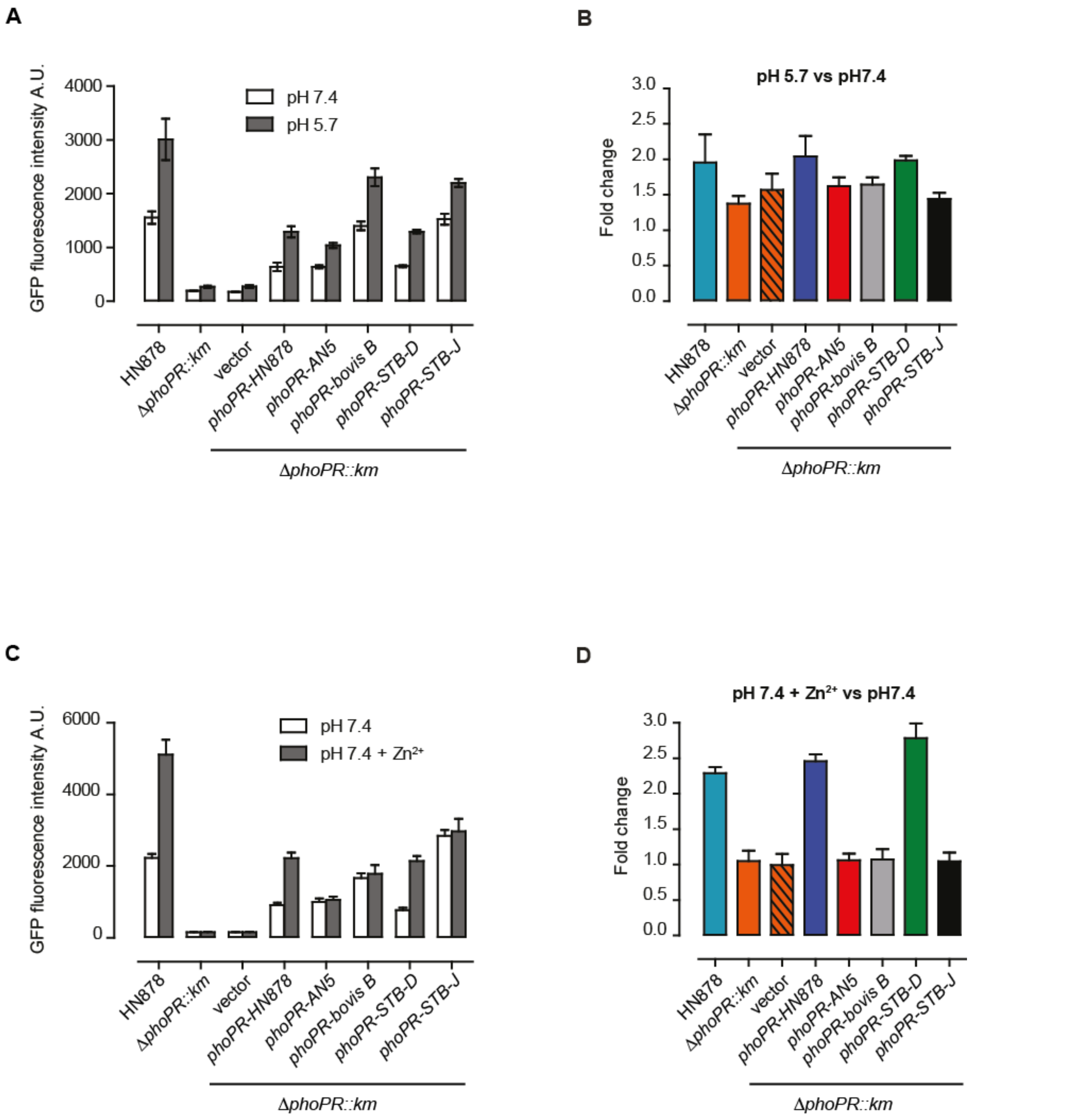

Supplementary Figure 4: GFP fluorescence at neutral, acidic pH or high Zn^{2+} concentration in recombinant HN878 strains expressing the *phoPR* variants and the fluorescent reporter.

Recombinant *M. tuberculosis* HN878 strains carrying different *phoPR* variants and the pEM12 fluorescent reporter were cultured under stress conditions (acidic pH or 500 µM $ZnSO_4$) or in non-stress control medium (pH 7.4). GFP fluorescence intensity under each condition is shown in panels **(A)** and **(C)**. The relative change in GFP fluorescence compared to the control condition is expressed as fold change in panels **(B)** and **(D).** Fluorescence was measured by flow cytometry from a gated population of 50,000 recorded events. Error bars represent the mean ± standard deviation from three independent experiments (n = 3).

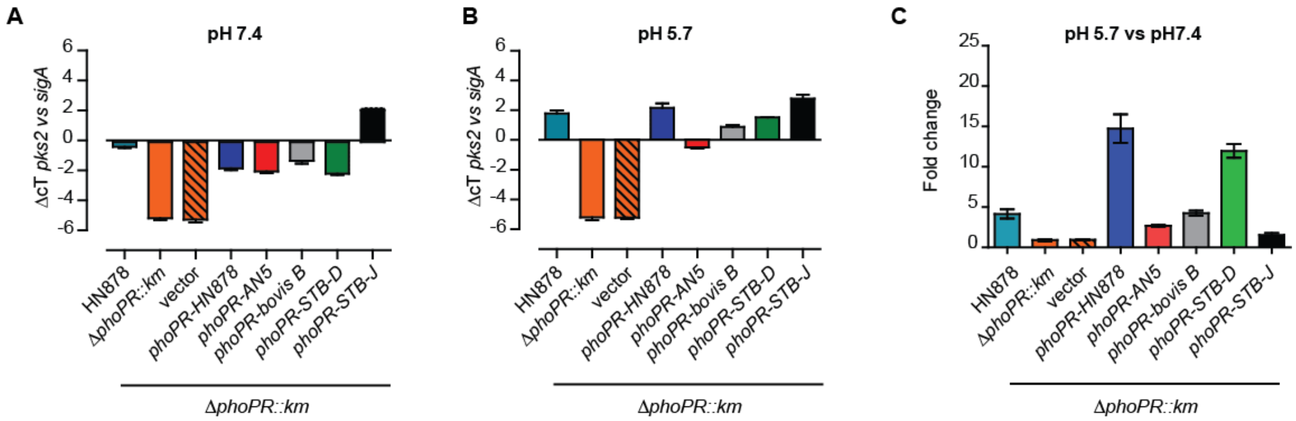

Supplementary Figure 5: Impact of PhoPR variants on *pks2* expression in response to pH. (A, B) Relative expression of the *pks2* gene, normalized to the housekeeping gene *sigA*, in strains expressing different *phoPR* variants under non-stress (pH 7.4; **panel A**) or acidic stress (pH 5.7; **panel B**) conditions. **(C)** Induction of *pks2* expression under acidic conditions is shown as fold change relative to expression at pH 7.4. Gene expression was quantified by RT-qPCR. Error bars represent the mean ± standard deviation of three technical replicates from a single experiment (n = 1).

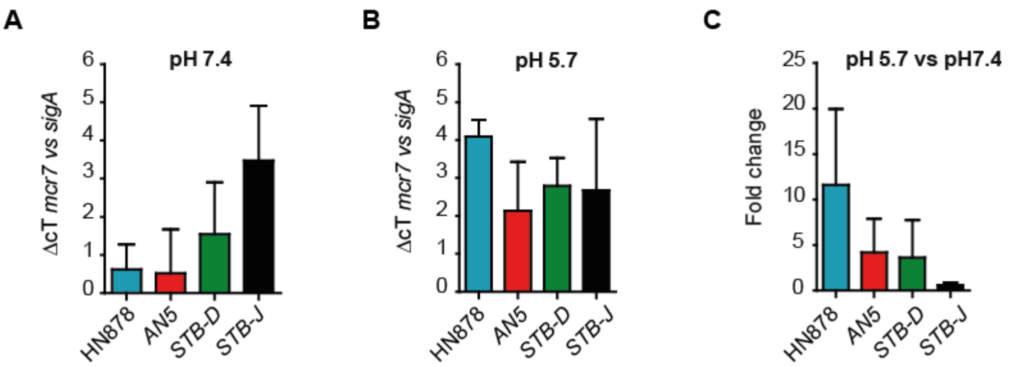

Supplementary Figure 6: Induction of *mcr7* by acidic pH in native strains.

(A, B) Relative expression of the *mcr7* gene, normalized to the housekeeping gene *sigA*, in native strains under non-stress (pH 7.4; **panel A**) or acidic stress (pH 5.7; **panel B**) conditions. **(C)** Induction of *mcr7* expression under acidic conditions is shown as fold change relative to expression at pH 7.4. Gene expression was quantified by RT-qPCR. Error bars represent the mean ± standard deviation of three technical replicates and three independent experiments (n = 3).

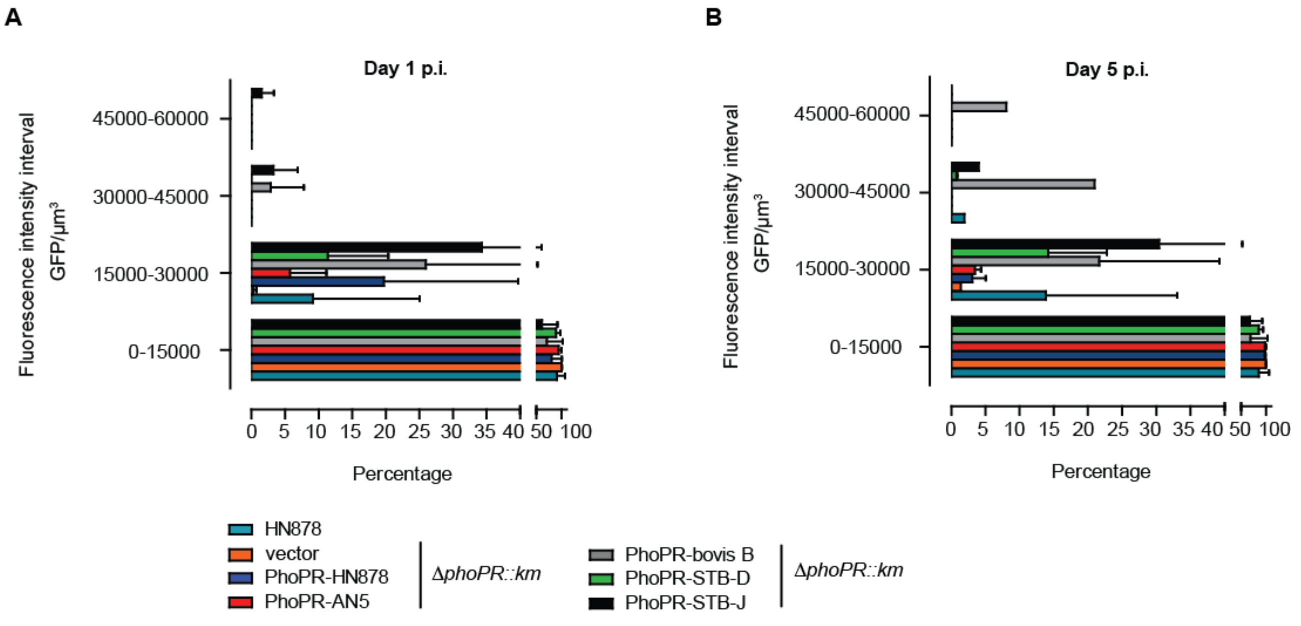

Supplementary Figure 7: GFP intensity in individual bacterial cell during infection of hMDAM. Quantification of GFP fluorescence in intracellular recombinant *M. tuberculosis* strains after 1 day and 5 days of infection. Monocytes isolated from the blood of three healthy donors were differentiated into alveolar macrophages and infected at a multiplicity of infection (MOI) of 2:1. Infected macrophages were visualized at one day post-infection (p.i.) **(A)** and five days p.i. **(B)** following labeling with CellTracker™ (BMQC) and fixation. Activation of the PhoPR regulon was assessed by measuring GFP (green) a pnd mCherry (red) signal per bacterium using fluorescence microscopy. For each strain, images were acquired from five distinct microscopic fields. Each bar represents the percentage of bacteria per range of GFP fluorescence intensity.

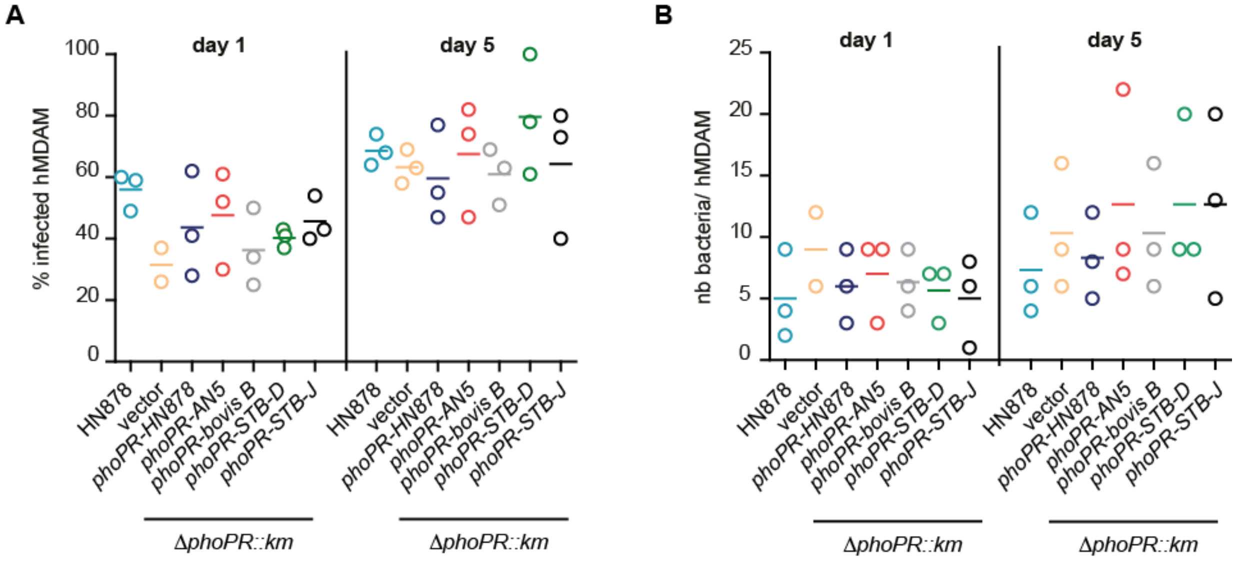

Supplementary Figure 8: Quantification of alveolar macrophage infection by fluorescence microscopy Infected alveolar macrophages from Figure 6 were visualized by fluorescent microscopy at one and five days p.i. following labeling with CellTracker™ (BMQC) and fixation. Infection of macrophages was manually assessed by quantifying both the percentage of cells having ingested at least one bacterium expressing mcherry **(A)** and the number of bacteria per infected cell **(B)** in the images. For each strain, images were acquired from five distinct microscopic fields. Data are presented as means ± SEM of 3 independent donors.

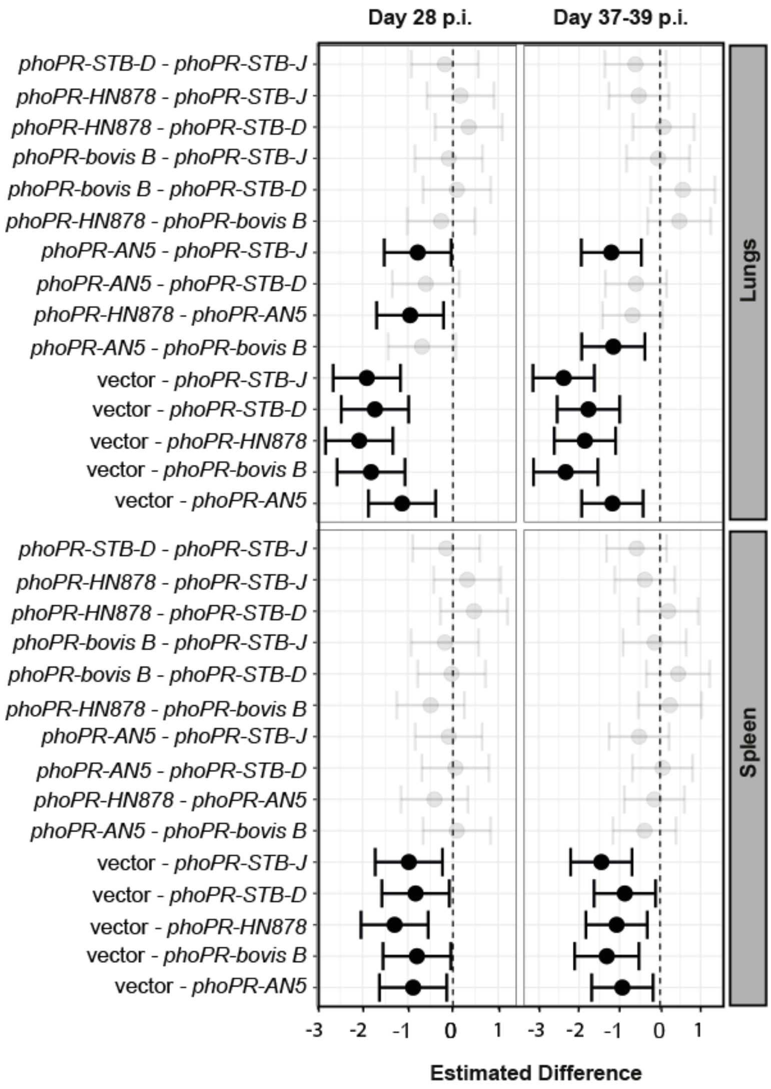

Supplementary Figure 9: Statistical analyses of the data from Figure 7, comparing results between pair of strains at days 28 and 37-39 p.i.

Statistical analyses were performed as described in Material and Methods. Estimated difference and 95% confidence intervals (CI) are shown. Comparisons for which the absence of difference falls outside the CI are therefore considered as significant (full-color traits vs. shaded traits, i.e. non-significant). Greater distance of the CI from the absence of difference implies higher significance.

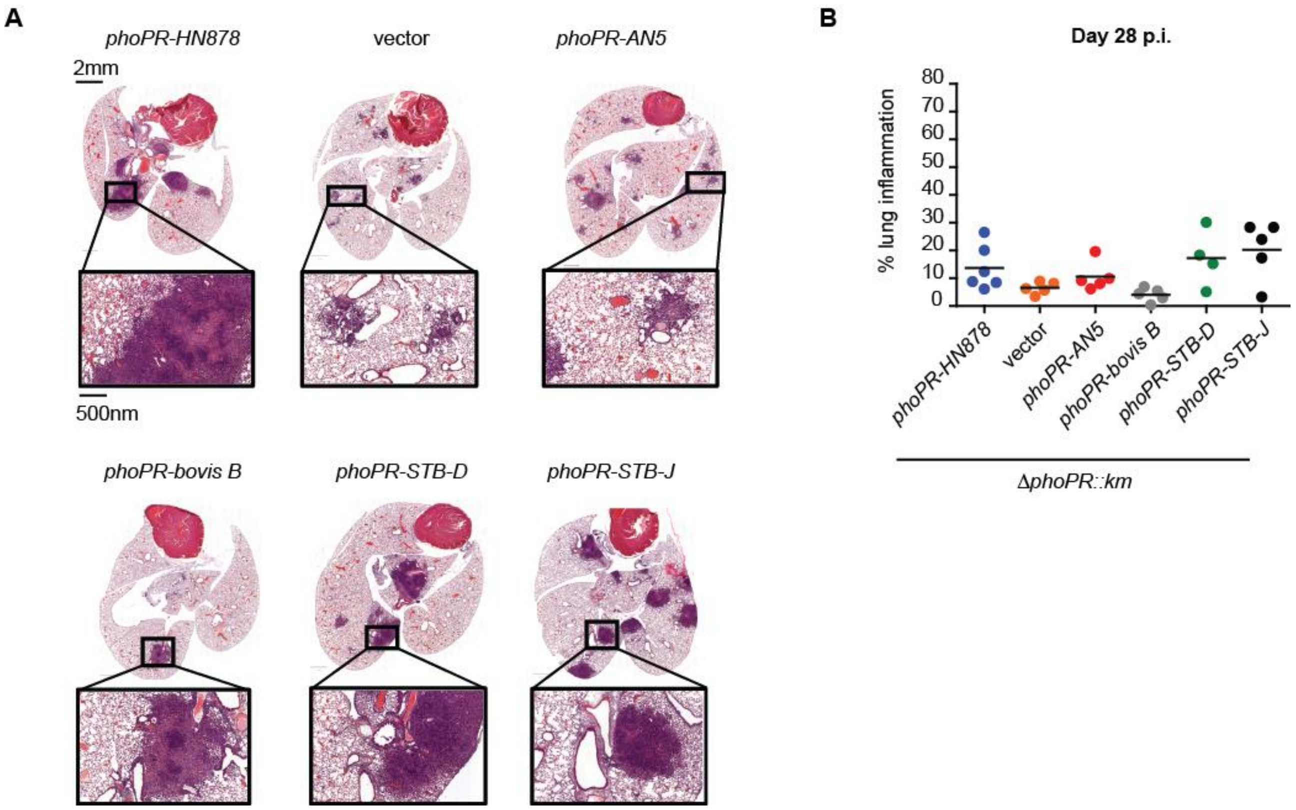

Supplementary Figure 10: Histopathological analysis of lung inflammation in the various groups of mice at 28 days post-infection.

C3HeB/FeJ mice were intranasally infected with six different recombinant *MTB* HN878 strains carrying distinct *phoPR* variants (n = 5 mice per strain; total n = 30). Pulmonary inflammation was assessed at 28 days post-infection. Lung tissues were formalin-fixed, paraffin-embedded, and stained with hematoxylin and eosin (H&E) to visualize histopathological lesions **(A)**. Interestingly, mice infected with the Δ*phoPR::km* mutant or the *phoPR-AN5* complemented strain showed a multitude of small lesions (≈17 lesions/lung) scattered throughout the lungs, whereas mice infected with the *phoPR-bovis-B* complemented strain displayed a lower number of lesions (≈5 lesions/lung), some of which reaching an approximately 5- to 15-fold larger size. The inflamed lung area was quantified as a percentage of total lung surface using QuPath software, based on manual delineation of inflamed regions **(B)**. Each data point represents an individual mouse. This experiment was performed once.

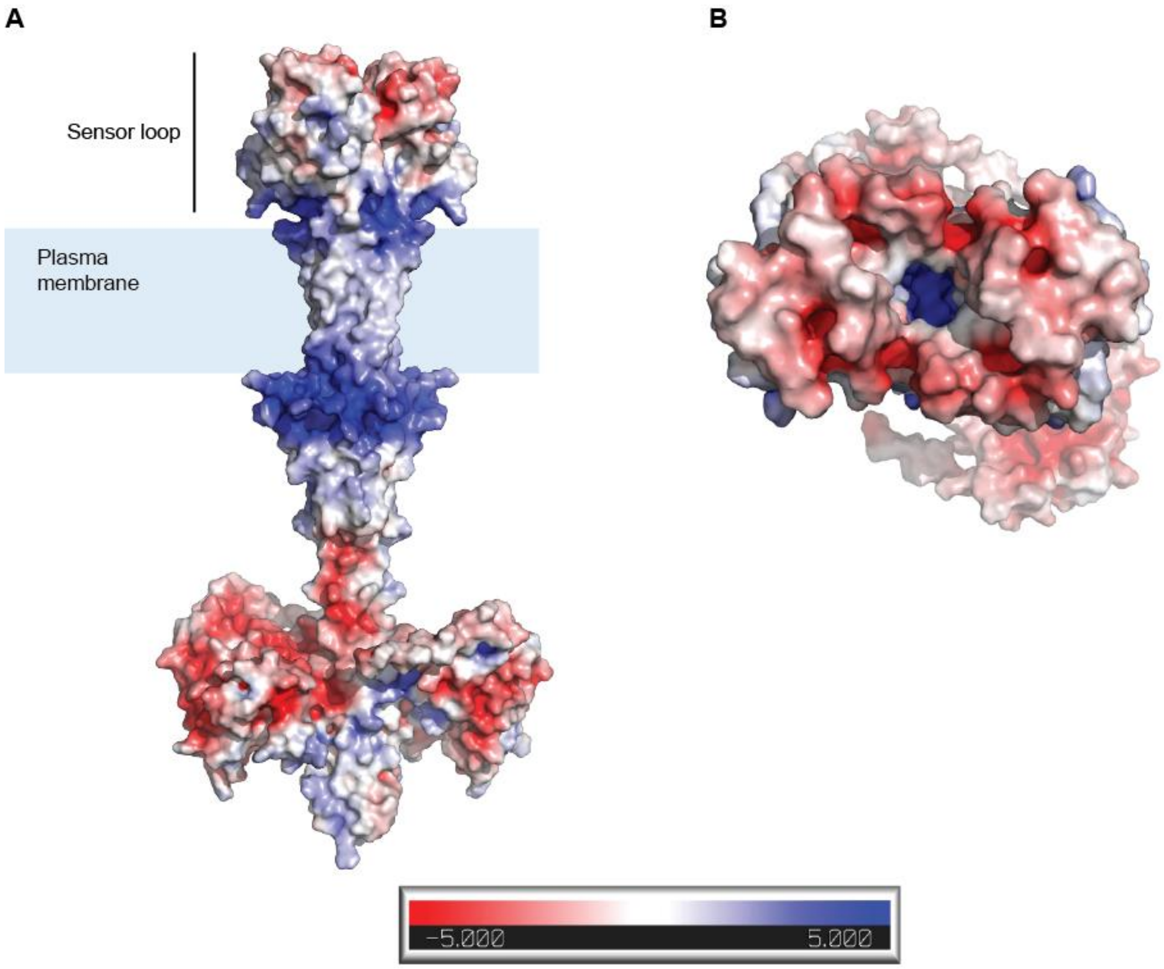

Supplementary Figure 11: AlphaFold-Multimer predicted structure of the *MTB* PhoR dimer with electrostatic potential highlighted.

The three-dimensional structure of the histidine kinase PhoR was predicted using AlphaFold-Multimer, and its electrostatic surface potential was computed in PyMOL from the predicted coordinates (1) . Negatively and positively charged regions are shown in red (−5 kBT/e) and blue (+5 kBT/e), respectively, while neutral areas appear in white.

(A) Front view of the PhoR dimer.

(B) Top view highlighting the sensor domain.

The electrostatic surface distribution may contribute to signal recognition or membrane association, and highlights potential regions involved in environmental sensing.

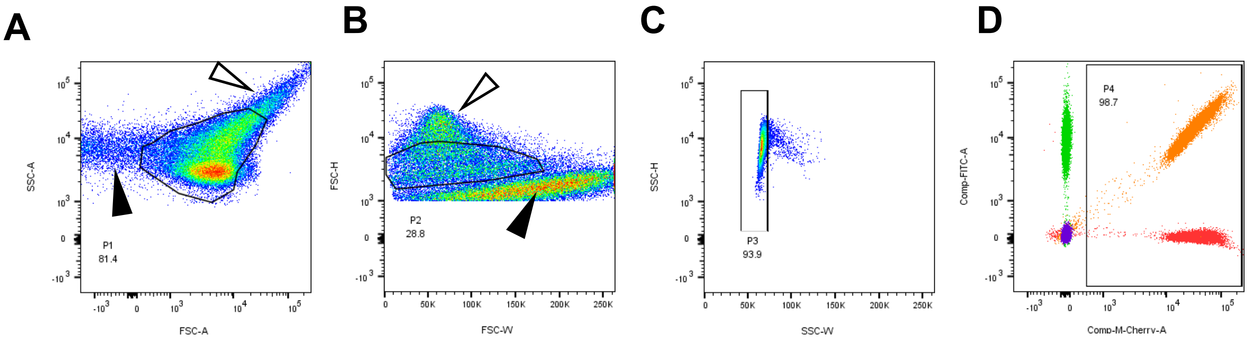

Supplementary Figure 12: Bacterial population selection strategy for flow cytometry analysis. For each strain, 50,000 bacteria were selected for analysis. Panels A–D illustrate the distribution of bacterial populations, with the color gradient from blue to red representing bacterial concentration, red indicating areas of highest density.

A. From the initial 50,000 bacteria, the first population (P1) was gated based on two parameters: side scatter (SSC), reflecting cellular complexity or granularity, and forward scatter (FSC), corresponding to cell size. This step excludes dead bacteria or debris (black arrow) as well as bacterial aggregates (white arrow) present in the sample.

B. A second, more stringent gating (P2) was applied on P1, based on FSC (size), to further remove dead cells/debris (black arrow) and aggregates (white arrow).

C. From P2, a third, even more refined population (P3) was selected based on SSC (granularity).

D. Overlay of distribution zones from control strains according to GFP and mCherry fluorescence intensity. Using control strains HN878 non-fluorescent (purple), HN878:mCherry (red), HN878:GFP (green), and HN878:GFP:mCherry (orange), the fluorescence distribution zones were established. A fourth gating (P4) was then defined to select all bacteria constitutively expressing mCherry and inducibly expressing GFP.

Supplementary Table 1: Name and main features of strains, plasmids used in this study.

Name	Relevant characteristics	Ref./Source
**Strains**		
HN878	*M. tuberculosis* (Lineage 2)	
Δ*phoPR::km*	PMM335: HN878, Δ*phoPR::km*	This study
**plasmids**		
pEM08	Integrative *E. coli*/mycobacteria shuttle vector derived from pMV361 carrying the integration system from bacteriophage L5 and a streptomycine resistance gene	This study,(11)
pEM09	pEM08 carrying the *phoPR* genes from *M. tuberculosis* HN878 (*phoPR-HN878*)	This study
pEM10	pEM08 carrying the *phoPR* genes from *M. bovis* AN5 (*phoPR-AN5)*	This study
pEM11	pEM08 carrying the *phoPR* genes from *M. canettii* STB-D (*phoPR-STB-D)*	This study
pEM14	pEM08 carrying the *phoPR* genes from *M. bovis* B (*phoPR-bovis-B)*	This study
pWM424	pEM08 carrying the *phoPR* genes from *M. canettii* STB-J (*phoPR-STB-J)*	This study
pEM12	Integrative *E. coli*/mycobacteria shuttle vector derived from pMV366H carrying the integration system from bacteriophage *ms6* and a hygromycine resistance gene	This study,(12)

Supplementary Table 2: Name and sequences of primers used in this study

Name	Sequence (5’->3’)
**Mutant or plasmid construction**	
EM1	CCAGCCTCGGTGTTTCAT
EM2	GTTCATGACCGGTGGTCGACCTGCTGAC
EM3	GAGCGGATACGACGTCACCGTGCGAGTC
EM4	GCAAGTCCGTCACCAATCC
EM5	GCCACCTGCGAGTTCGC
EM6	CCTTCTTCGACACACACATTG
EM7	GTCGACCACCGGTCATGAACAATAAAACTGTCTGC
EM8	CGGTGACGTCGTATCCGCTCATGAATTAATTCTTAG
KmF	GCCATCCTATGGAACTGCC
KmR	GCCTAGAGCAAGACGTTTCC
R1	GCCAGACACCTTCGAGGAA
R2	GTCGGTGATGAACTGTCGC
EM23	CATCCCGTCGGGTTTGATCTTGACCGTTATCGGAAGCG
EM28	CATCCCGTCGGGTTTAAAC CAAGCATCAGCCGAAACATCGTCAG
EM29	CATCCCGTCGGGTTTAAAC GATCTTGACCGTTATCGGAAGCG
EM30	GTCCTGGCTGGGTTTAAACCGTGATAAGCGCAGGCCAAGATCAG
EM31	GTCCTGGCTGGGTTTAAACACATCGAAGCGCAGGCCAAGATCAG
EM32	GTCCTGGCTGGGTTTAAACGCCTAAAAGCGCAGGCCAAGATCAG
EM33	GTCCTGGCTGGGTTTAAACCAGACTAAGCGCAGGCCAAGATCAG
EM34	CATCCCGTCGGGTTTAAACTGGCGCTCAACGGAATGCTG
EM35	GTCCTGGCTGGGTTTAAACCATTCAAAGCGCAGGCCAAGATCAG
orim	GGGATTACACATGACCAACTTC
**RT-qPCR analyses**	
RT-sigA-Fw	CCGATGACGACGAGGAGATC
RT-sigA-Rv	CGGAGGCCTTGTCCTTTTC
RT-mcr7-Fw	ACGCCGCGAGGACATG
RT-mcr7-Rv	AGGGAGCTGCTTGGACAGAA
RT-pks2-Fw	GCATCGGTGAAGACCAACTTC
RT-pks2-Rv	GATTACGTGGAACCACACCATGT
RT-phoR-Fw	TCGAGGAAGCCCAAATCTGG
RT_phoR-Rv	ATGGCAGTGTTGTCGTTGAG
RT-phoP-Fw	GTGACGACTATGTGACAAAGCC
RT-phoP-Rv	TTACGTGGTTCCTTGTTGCC

References:

1. Malaga W, Payros D, Meunier E, Frigui W, Sayes F, Pawlik A, Orgeur M, Berrone C, Moreau F, Mazeres S, Gonzalo-Asensio J, Rengel D, Martin C, Astarie-Dequeker C, Mourey L, Brosch R, Guilhot C. 2023. Natural mutations in the sensor kinase of the PhoPR two-component regulatory system modulate virulence of ancestor-like tuberculosis bacilli. PLoS pathogens 19:e1011437.

2. Schreuder LJ, Carroll P, Muwanguzi-Karugaba J, Kokoczka R, Brown AC, Parish T. 2015. *Mycobacterium tuberculosis* H37Rv has a single nucleotide polymorphism in PhoR which affects cell wall hydrophobicity and gene expression. Microbiology 161:765–773.

3. Gonzalo-Asensio J, Malaga W, Pawlik A, Astarie-Dequeker C, Passemar C, Moreau F, Laval F, Daffé M, Martin C, Brosch R, Guilhot C. 2014. Evolutionary history of tuberculosis shaped by conserved mutations in the PhoPR virulence regulator. Proc Natl Acad Sci USA 111:11491–11496.

4. Soto CY, Menendez MC, Perez E, Samper S, Gomez AB, Garcia MJ, Martin C. 2004. IS*6110* mediates increased transcription of the *phoP* virulence gene in a multidrug-resistant clinical isolate responsible for tuberculosis outbreaks. 1. J Clin Microbiol 42:212–9.

5. Rivero A, Marquez M, Santos J, Pinedo A, Sanchez MA, Esteve A, Samper S, Martin C. 2001. High rate of tuberculosis reinfection during a nosocomial outbreak of multidrug-resistant tuberculosis caused by *Mycobacterium bovis* strain B. Clin Inf Dis 32:159–61.

6. Supply P, Marceau M, Mangenot S, Roche D, Rouanet C, Khanna V, Majlessi L, Criscuolo A, Tap J, Pawlik A, Fiette L, Orgeur M, Fabre M, Parmentier C, Frigui W, Simeone R, Boritsch E, Debrie A-S, Willery E, Walker D, Quail MA, Ma L, Bouchier C, Salvignol G, Sayes F, Cascioferro A, Seemann T, Barbe V, Locht C, Gutierrez M-C, Leclerc C, Bentley SD, Stinear TP, Brisse S, Médigue C, Parkhill J, Cruveiller S, Brosch R. 2013. Genomic analysis of smooth tubercle bacilli provides insights into ancestry and pathoadaptation of *Mycobacterium tuberculosis*. Nat Genet 45:172–179.

7. Blouin Y, Cazajous G, Dehan C, Soler C, Vong R, Hassan MO, Hauck Y, Boulais C, Andriamanantena D, Martinaud C, Martin E, Pourcel C, Vergnaud G. 2014. Progenitor "*Mycobacterium canettii*" clone responsible for lymph node tuberculosis epidemic, Djibouti. Emerg Inf Dis 20:21–28.

8. Koeck J-L, Fabre M, Simon F, Daffé M, Garnotel AM, Matan AB, Gerome P, Bernatas JJ, Buisson Y, Pourcel C. 2011. Clinical characteristics of the smooth tubercle bacilli "*Mycobacterium canettii*" infection suggest the existence of an environmental reservoir. Clin Microbiol Infect 17:1013–1019.

9. van Kessel JC, Hatfull GF. 2008. Mycobacterial recombineering. Methods Mol Biol 435:203–215.

10. Abramovitch RB, Rohde KH, Hsu F-F, Russell DG. 2011. *aprABC*: a *Mycobacterium tuberculosis* complex-specific locus that modulates pH-driven adaptation to the macrophage phagosome. Mol Microbiol 80:678–694.

11. C. K. Stover, V. F. de la Cruz, T. R. Fuerst, J. E. Burlein, L. A. Benson, L. T. Bennett, G. P. Bansal, J. F. Young, M. H. Lee, G. F. Hatfull, S. B. Snapper, R. G. Barletta, W. R. Jacobs Jr, B. R. Bloom. 1991. New use of BCG for recombinant vaccines. Nature 351:456–460.

12. E. Anes, I. Portugal, J. Moniz-Pereira. 1992. Insertion into the *Mycobacterium smegmatis* genome of the *aph* gene through lysogenization with the temperate mycobacteriophage Ms6. FEMS Microbiology Letters 95:21–26.
